# Supplementary material for: H3K4 Methylation-Dependent Memory of Somatic Cell Identity Inhibits Reprogramming and Development of Nuclear Transfer Embryos
Source: Cell Stem Cell. 2017 Jul 6;21(1):135–143.e6. doi: 10.1016/j.stem.2017.03.003 (PMC5505866; doi:10.1016/j.stem.2017.03.003)
Supplement: Document S1. Figures S1–S4 and Tables S1–S4 [file mmc1.pdf]

Cell Stem Cell, Volume *21*

Supplemental Information

H3K4 Methylation-Dependent Memory of Somatic Cell Identity Inhibits Reprogramming and Development of Nuclear Transfer Embryos

Eva Hörmanseder, Angela Simeone, George E. Allen, Charles R. Bradshaw, Magdalena Figlmüller, John Gurdon, and Jerome Jullien

Figure S1

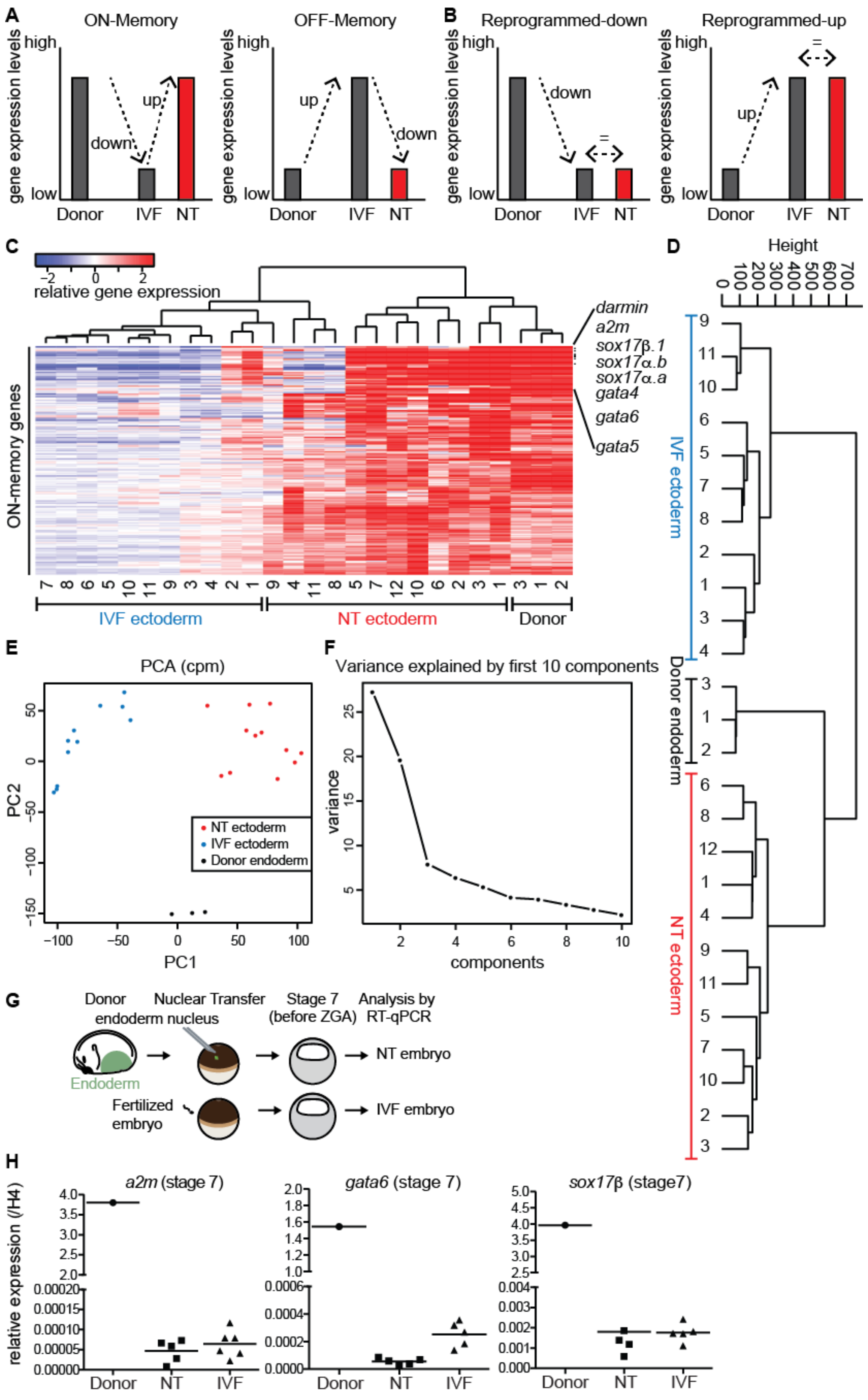

Figure S1. Related to Figure 1; Changes from donor endoderm cells to NT embryos ectoderm cells: Memory and reprogramming of gene expression. (A) The gene expression in the donor endoderm cells was compared with gene expression in the ectoderm cells of control IVF embryos. This revealed genes that are differentially expressed between the two cell-types. Next, gene expression between the ectoderm cells of control IVF embryos and the ectoderm cells of NT embryos was compared. This revealed genes that are differentially expressed between IVF and NT

ectoderm cells and thus represent reprogramming resistant genes. The group of reprogramming resistant genes comprises ON-memory genes, which are genes that were expressed in the endoderm donor cells and are down-regulated in ectoderm cells of IVF embryos, but remain up-regulated in the ectoderm cells of NT embryos. Furthermore, the group of reprogramming resistant genes also contains OFF-memory genes. These are genes that are up-regulated in IVF ectoderm when compared to endoderm donor cells, but remain down-regulated in NT ectoderm cells. **(B)** Instead genes that are differentially expressed between the endoderm donor cells and the IVF ectoderm cells and that were similarly expressed in the IVF and NT ectoderm cells represent successfully reprogrammed genes. **(C)** All NT embryos show genes with an active state of gene-expression (ON-memory). Heatmap illustration comparing ON-memory(3FC) gene expression in ectoderm tissues of single (not pooled) IVF and NT embryos as well as donor endoderm cells. Rows and columns are sorted by hierarchical clustering (agglomeration method: complete, Euclidian distance function). Examples of endoderm lineage genes showing ON-memory are indicated. **(D-F)** Filtered and normalized RNAseq data of single ectoderm tissues of IVF and NT embryos as well as donor endoderm cells presented in Fig.1 and 2. **(D)** Hierarchical transcriptome clustering analysis (agglomeration method: Ward.D as implemented in R, Euclidean distance function) **(E)** Principal component analysis (PCA). First two principal components (which explain 27% and 16.5% of the variance) were computed using the R function prcomp() with the parameter cor = T. **(F)** Percentage of variance explained by the first 10 principal components of data shown in (E). (G) Endoderm donor specific genes are not detected before zygotic genome activation – design of NT experiments. After NT of an endoderm donor nucleus to an enucleated egg, stage 7 embryos (prior to zygotic genome activation, ZGA) were collected. As controls, eggs were fertilized and collected at the same stage. (H) Donor endoderm-cells as well as NT and IVF ectoderm cells were analysed by RT-qPCR for *a2m*, *gata6* and *sox17β* relative to *H4* in whole stage 7 embryos. NT, nuclear transfer; IVF, *in vitro* fertilized; RT-qPCR, quantitative real-time PCR;

Figure S2

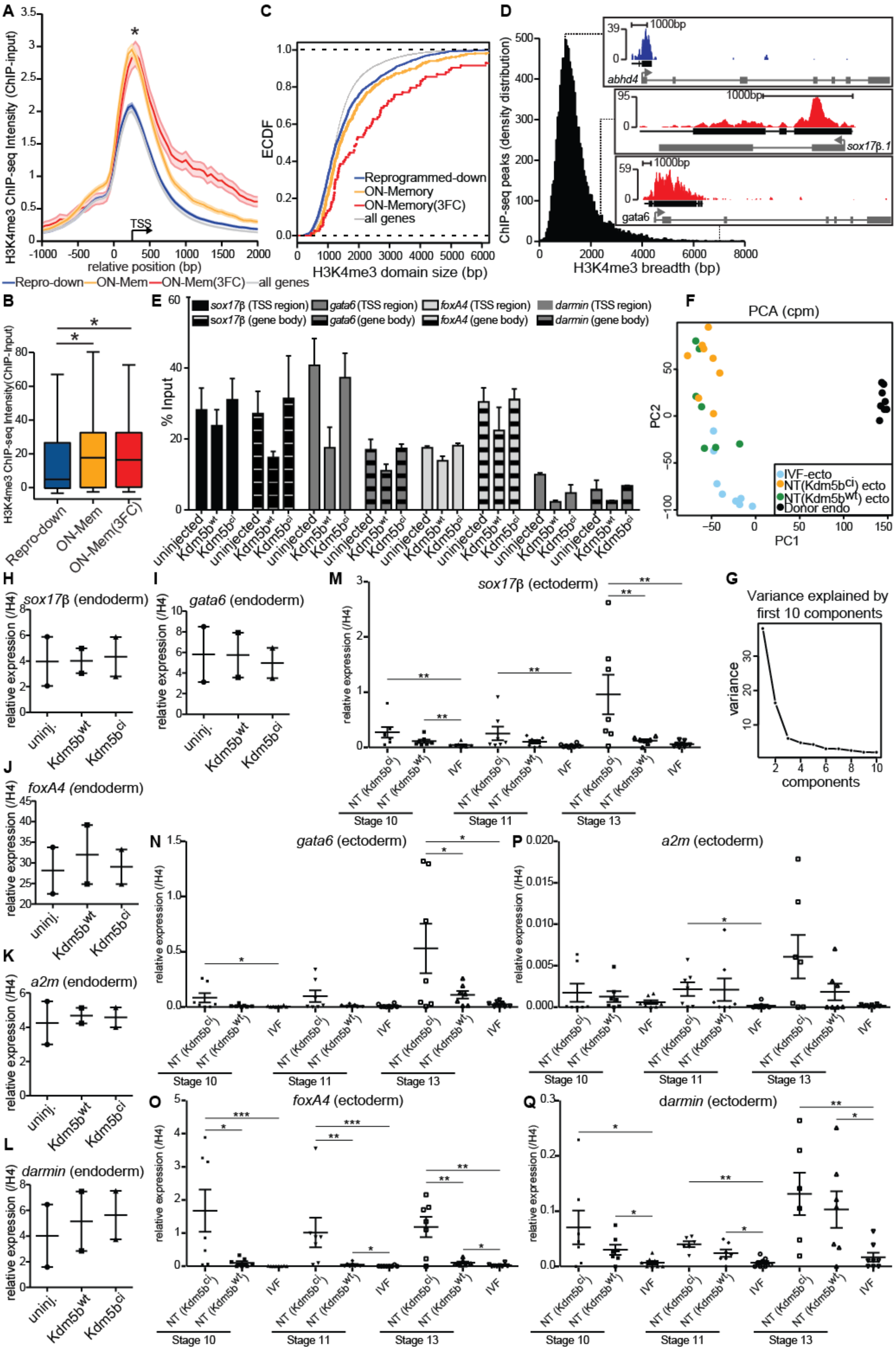
A
H3K4me3 ChIP-seq Intensity (ChIP-input)
relative position (bp)
TSS
Repro-down ON-Mem ON-Mem(3FC) all genes
C
ECDF
H3K4me3 domain size (bp)
Reprogrammed-down
ON-Memory
ON-Memory(3FC)
all genes
D
ChIP-seq peaks (density distribution)
H3K4me3 breadth (bp)
1000bp
abhd4
sox17β.1
gata6
B
H3K4me3 ChIP-seq Intensity(ChIP-Input)
Repro-down
ON-Mem
ON-Mem(3FC)
E
sox17β (TSS region)
sox17β (gene body)
gata6 (TSS region)
gata6 (gene body)
foxA4 (TSS region)
foxA4 (gene body)
darmin (TSS region)
darmin (gene body)
% Input
uninjected
Kdm5bwt
Kdm5bci
F
PCA (cpm)
PC1
PC2
IVF-ecto
NT(Kdm5bci) ecto
NT(Kdm5bwt) ecto
Donor endo
G
Variance explained by first 10 components
variance
components
H
sox17β (endoderm)
I
gata6 (endoderm)
J
foxA4 (endoderm)
K
a2m (endoderm)
L
darmin (endoderm)
relative expression (/H4)
uninj.
Kdm5bwt
Kdm5bci
M
sox17β (ectoderm)
N
gata6 (ectoderm)
O
foxA4 (ectoderm)
P
a2m (ectoderm)
Q
darmin (ectoderm)
NT (Kdm5bci)
NT (Kdm5bwt)
IVF
Stage 10
Stage 11
Stage 13

Figure S2. Related to Figure 2 and 3; ON-memory genes are enriched for H3K4me3 when compared to reprogrammed-down genes in *Xenopus* endoderm donor cells and Kdm5b treatment of donor reduces ON-memory gene expression in the resulting NT embryos.

(A-D) H3K4me3 ChIP-seq data was generated from endoderm cells of neurula-stage embryos as used for NT experiments; second biological replicate is shown here. Read counts are normalized by input and total mapped reads. **(A)** TSS metaplot of the average intensity of H3K4me3 modifications in endoderm cells are shown for reprogrammed-down, ON-memory genes, ON-memory(3FC) and all genes from the *Xenopus* genome. ON-memory(3FC) and ON-memory ChIP-seq intensities are higher when compared to reprogrammed-down genes (p-value= 0.071 and *p-value= 0.0006, respectively; 4 kb window, KS-test). **(B)** ON-memory genes when compared to reprogrammed-down genes, show increased H3K4me3 levels in the donor cells. Box plot comparing mean H3K4me3 ChIP-seq intensities of reprogrammed-down, ON-memory and ON-memory(3FC) in a 4kb window centred on the TSS (*p-value< 0.001, KS-test). **(C)** Empirical cumulative distribution function comparing H3K4me3 domain size around the TSS of reprogrammed-down, ON-memory genes, ON-memory(3FC), and all genes from the *Xenopus* genome. ON-memory(3FC) genes show a significant increase in H3K4me3 breadth when compared to reprogrammed-down genes (p-value= 8.55E-07, KS-test; ChIP-seq peaks called by MACS2). **(D)** Breadth distribution of H3K4me3 ChIP-seq peaks called by MACS2. Inserts are examples of H3K4me3 regions of a reprogrammed-down gene (*abhd4*) and two ON-memory(3FC) genes, *sox17β.1* and *gata6* (NM_001087983.1). **(E)** ChIP-RTqPCR verification of the reduction in H3K4me3 levels upon $Kdm5b^{wt}$ treatment on candidate ON-memory genes. ChIP-RTqPCR showing H3K4me3 enrichment over *sox17β*, *gata6*, *foxa4* and *darmin* TSS and gene body regions in endoderm cells isolated from uninjected, $Kdm5b^{wt}$ and $Kdm5b^{ci}$ expressing stage 18 embryos (n=2). Data are presented as mean ± SEM. TSS, transcriptional start site. **(F)** Principal component analysis (PCA) of filtered and normalized RNAseq data of single ectoderm tissues of IVF and NT embryos as well as donor endoderm cells, see Fig.3. First two principal components (which explain 27% and 16.5% of the variance) were computed using the R function prcomp() with the parameter cor = T. **(G)** Percentage of variance explained by the first 10 principal components of data shown in (F). **(H-Q)** Reduction of H3K4 methylation via $Kdm5b^{wt}$ in donor cells via expression of $H3.3^{K4M}$ reduces expression of some ON-memory genes in the resulting NT embryos throughout gastrulation. In two independent experiments, the expression of candidate memory genes (*sox17β*, *gata6*, *foxa4*, *a2m* and *darmin*) was assessed by RT-qPCR in **(H-L)** the endoderm donor cells and **(M-Q)** in the ectoderm cells of 7 NT($Kdm5b^{ci}$) , 7 NT($Kdm5b^{wt}$) and 8 IVF embryos (single, not pooled) at different stages during gastrulation. Increased candidate memory gene expression can be observed in all treatment control NT($Kdm5b^{ci}$) embryos when compared to the IVF–embryos. Candidate ON-memory gene expression is reduced upon treatment of the donor cell with $Kdm5b^{wt}$ and for some genes this effect is more pronounced (*sox17β*, *gata6*,*foxa4*) than for others (*a2m* and *darmin*). * p ≤ 0.05, ** p ≤ 0.01, *** p ≤ 0.001; data are presented as mean ± SEM. Box plots: middle line in the box indicates the median, the box edges indicate the 25th/75th percentiles, the whiskers indicate the min and max.

Figure S3

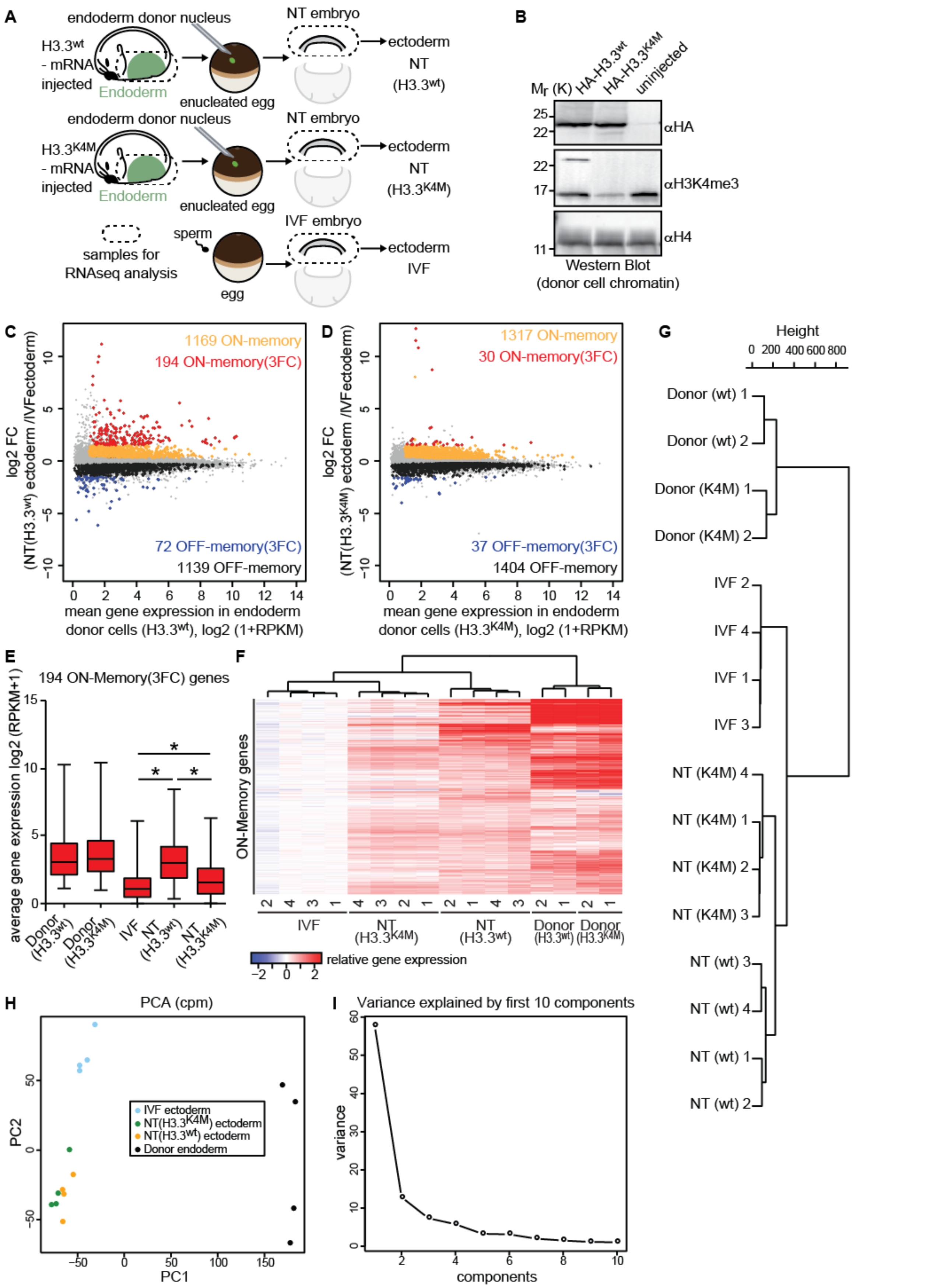

Figure S3. Related to Figure 3; Inhibition of H3K4me3 specific methyltransferases in the donor cells via expression of H3.3^{K4M} reduces ON-memory gene expression in the resulting NT embryos. (A) Design of NT experiments. After NT of an endoderm donor nucleus (expressing H3.3^{K4M} or H3.3^{wt}) to an enucleated egg, gastrula embryos were collected. As controls, IVF

embryos were collected at the same stage. The endoderm was isolated from donor embryos, the ectoderm was isolated from NT and IVF embryos, and all tissues were analysed by RNA-seq. **(B)** Western Blot analysis showing that H3.3^{K4M}, but not to H3.3^{wt} expression reduces H3K4me3 levels to ≈75% of control (uninjected) levels in neurula stage embryos. **(C-F)** H3.3^{K4M} expression in the donor cells reduces the number of miss-regulated genes in NT embryos when compared to IVF embryos. MA plot comparing gene expression between ectoderm cells of **(C)** NT(H3.3^{wt}) and IVF embryos or **(D)** NT(H3.3^{K4M}) and IVF embryos. The average log2 fold change in expression of transcripts in ectoderm cells of NT embryos over IVF embryos was plotted on the y axis, the mean log2 (1+RPKM) gene expression in the endoderm donor cells was plotted on the x axis (ectoderm of 4 NT(H3.3^{K4M}), 4 NT(H3.3^{wt}) and 4 IVF embryos; 2 endoderm tissues of H3.3^{K4M} - or H3.3^{wt} - expressing embryos. n=1, see Tab.S1). Gray, all identified transcripts; orange, ON-memory and black, OFF-memory; red, ON-memory genes; blue, OFF-memory genes. **(E)** Box plots comparing the mean expression levels (RPKM) of ON-memory transcripts in endoderm donor cells and the ectoderm tissues of IVF , NT(H3.3^{wt}) and NT(H3.3^{K4M}) embryos. (*p-values<0.001) **(F)** Heatmap illustration comparing ON-memory gene expression in single ectoderm tissues of IVF, NT(H3.3^{K4M}) and NT(H3.3^{WT}) embryos as well as in the endoderm donor cells. Rows and columns are sorted by hierarchical clustering. For detailed numbers see Table S5. **(G)** Hierarchical transcriptome clustering analysis (agglomeration method: Ward.D as implemented in R, Euclidean distance function) and **(H)** Principal component analysis (PCA) of filtered and normalized RNAseq data of single ectoderm tissues of IVF and NT embryos as well as donor endoderm cells presented in this figure. First two principal components (which explain 27% and 16.5% of the variance) were computed using the R function prcomp() with the parameter cor = T. **(I)** Percentage of variance explained by the first 10 principal components of data shown in (H).
Box plots: middle line in the box indicates the median, the box edges indicate the 25th/75th percentiles, the whiskers indicate the min and max.

Figure S4

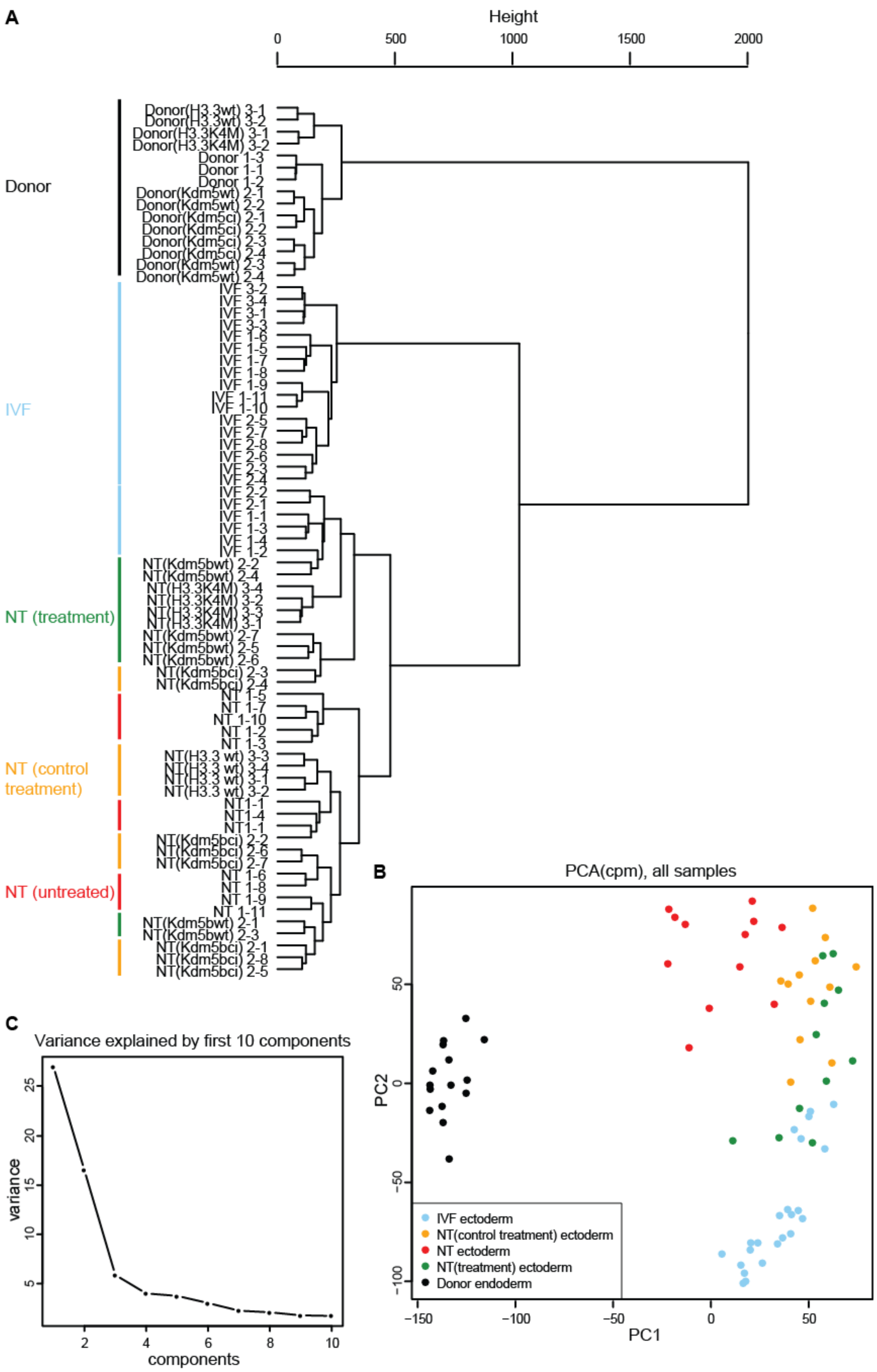

Figure S4. Related to Fig.1-4; Experimental variability analysis of RNAseq data presented in this study. (A) hierarchical clustering analysis performed on all experiments, filtered and normalized data. Colors codify the nature of the experiments: Donor cells, black; IVF, blue; NT

treatment ($Kdm5b^{wt}$ or $H3.3^{K4M}$), green; NT control ($Kdm5b^{ci}$ or $H3.3^{wt}$), orange; NT, red. In general, all RNA-seq experiments taken together group as expected into three classes (Donor, IVF and NT) irrespectively of the experimental batch. The hierarchical clustering was performed by using the Euclidean distance and ward.D linkage (as implemented in R). **(B)** First two principal components (which explain 27% and 16.5% of the variance, see panel (C)) of all experiments, filtered and normalized data. Also here, all RNA-seq experiments taken together group as expected into three classes (Donor, IVF and NT) irrespectively of the experimental batch. The PCA analysis was performed using the R function prcomp() using the parameter cor = T. Colors codify the nature of the experiments: Donor cells, black; IVF, blue; NT treatment($Kdm5b^{wt}$ or $H3.3^{K4M}$), green; NT control ($Kdm5b^{ci}$ or $H3.3^{wt}$), orange; NT, red. **(C)** Percentage of variance explained by the first 10 components.

Table S1. Related to Fig.1, 2, 3, S3 and 4; Overview of biological replicates.

Differential gene expression analyses								
**Embryo (tissues) analysed by RNAseq**	**NT Experiments (Fig.1 and 2)**			**Kdm5b NT Experiments (Fig.3)**			**H3.3 NT Experiments (Fig.S4)**	
	**Exp.1**	**Exp.2**	**Exp.3**		**Exp.1**	**Exp.2**		**Exp.1**
**Donor (endoderm)**	1	1	1	**Kdm5b^{wt}**	2	2	**H3.3^{K4M}**	2
				**Kdm5b^{ci}**	2	2	**H3.3^{wt}**	2
**NT (ectoderm)**	3	4	5	**Kdm5b^{wt}**	4	3	**H3.3^{K4M}**	4
				**Kdm5b^{ci}**	4	4	**H3.3^{wt}**	4
**IVF (ectoderm)**	3	4	4		4	4		4

H3K4 demethylation in donor nuclei improves embryonic development of NT-embryos (results of quantifications)			
**Developmental stage**	**NT (Kdm5b^{wt})**	**NT (Kdm5b^{ci})**	**IVF**
**NT**	677	687	N/A
**Cleaved (stage 10)**	56 (100%)	69 (100%)	152 (100%)
**Gastrulae (stage 11)**	54 (97%)	67 (97%)	152 (100%)
**Neurulae (stage 21)**	42 (79%)	50 (70%)	140 (92%)
**Feeding tadpole (stage 45)**	34 (60%)	24 (33%)	127 (84%)

Donor, Endoderm donor cells; NT, nuclear transfer embryo; IVF, *in vitro* fertilized embryo; Exp., experiment; Numbers represent the number of tissues sequenced individidually, not in pools. 1 tissue was harvested from 1 embryo.

Table S2. Related to Fig.1 and 2; Overview of gene expression changes following nuclear transfer.

Categories	number of transcripts	% of total number of transcripts	Filters applied
**total number of transcripts**	24215	100%	CPM>1 in either all of the Donor- or 8 of IVF- or 8 of NT- samples.
**DE Donor and IVF**	17587	72.6%	FDR$^{Donor/IVF}$<0.05
**DE Donor and IVF and DE NT and IVF**	4504	18.6%	FDR $^{Donor/IVF}$<0.05 & FDR$^{NT/IVF}$ <0.05
**ON-Memory** (down-regulated in IVF vs Donor, up-regulated in NT vs IVF)	1534	6.3%	FDR$^{Donor/IVF}$<0.05 & logFC$^{Donor/IVF}$>0 & FDR$^{NT/IVF}$<0.05 & logFC$^{NT/IVF}$>0 & RPKM^{Donor}>1
**ON-Memory(3FC)** (down-regulated in IVF vs Donor, >3-fold up-regulated in NT vs IVF)	264	1.1%	FDR$^{Donor/IVF}$<0.05 & logFC$^{Donor/IVF}$>0 & FDR$^{NT/IVF}$<0.05 & logFC$^{NT/IVF}$>1.5 & RPKM^{Donor}>1
**OFF-Memory** (up-regulated in IVF vs Donor, down-regulated in NT vs IVF)	1346	5.6%	FDR$^{Donor/IVF}$<0.05 & logFC$^{Donor/IVF}$<0 & FDR$^{NT/IVF}$<0.05 & logFC$^{NT/IVF}$<0
**OFF-Memory(3FC)** (up-regulated in IVF vs Donor, >3-fold down-regulated in NT vs IVF)	88	0.36%	FDR$^{Donor/IVF}$<0.05 & logFC$^{Donor/IVF}$<0 & FDR$^{NT/IVF}$<0.05 & logFC$^{NT/IVF}$<-1.5
**Reprogrammed** (up- or down-regulated in IVF vs Donor, not DE between IVF and NT)	13083	54%	FDR$^{Donor/IVF}$<0.05 & exclude transcripts FDR$^{NT/IVF}$<0.05
**Reprogrammed-down** (down-regulated in IVF and NT vs Donor, not DE between IVF and NT)	6321	26.1%	FDR$^{Donor/IVF}$<0.05&logFC$^{Donor/IVF}$>0 &FDR$^{Donor/NT}$<0.05 & logFC$^{Donor/INT}$>0&RPKM^{Donor}>1&excludeFDR$^{NT/IVF}$<0.05
**Reprogrammed-up** (up-regulated in IVF and NT vs Donor, not DE between IVF and NT)	5501	22.7%	FDR$^{Donor/IVF}$<0.05 & logFC$^{Donor/IVF}$<0 &FDR$^{Donor/NT}$<0.05 & ogFC$^{Donor/IVF}$<0 & FDR$^{NT/IVF}$>0.05

Donor, Endoderm donor cells; NT, ectoderm of Nuclear transfer embryo, IVF, Ectoderm of *in vitro* fertilized embryo; DE, differentially expressed; FC, fold change; CPM, counts per million; FDR, false discovery rate; logFC, log2 fold change; RPKM, reads per kilobase per million;

Table S3. Related to Fig.3 and S3; Differential gene expression analysis of H3K4 demethylation experiments.

Overview of gene expression changes in H3K4 demethylated donor cells ($Kdm5b^{wt}$ or $H3.3^{K4M}$ mRNA injected) or control donor cells ($Kdm5b^{ci}$ or $H3.3^{wt}$ mRNA injected)

Categories	$Kdm5b^{ci}$ or $Kdm5b^{wt}$ treatment of donor cells		$H3.3^{wt}$ or $H3.3^{K4M}$ treatment of donor cells		Filters applied
	number of transcripts	% of ID transcripts	number of transcripts	% of ID transcripts	
**ID transcripts**	24758	100%	19210	100%	CPM>1 in either all of the donor, all IVF or all of NT samples
**DE Donor (treatment) vs Donor (control)**	102	0.4%	2953	15.4%	FDR<0.05
**total number of transcripts**	23318	99.6%	16257	84.6%	not DE in Donor($Kdm5b^{wt)}$ vs Donor($Kdm5b^{ci}$)

Overview of gene expression changes following nuclear transfer of H3K4 demethylated donor cells ($Kdm5b^{wt}$ or $H3.3^{K4M}$ treatment) or control donor cells ($Kdm5b^{ci}$ or $H3.3^{wt}$ treatment)

Categories	$Kdm5b^{ci}$ treatment of donor cells		$Kdm5b^{wt}$ treatment of donor cells		$H3.3^{wt}$ treatment of donor cells		$H3.3^{K4M}$ treatment of donor cells	
	number of transcripts	% of total transcripts	number of transcripts	% of total transcripts	number of transcripts	% of total transcripts	number of transcripts	% of total transcripts
**total number of transcripts**	23318	100%	23318	100%	16257	100%	16257	100%
**DE Donor and IVF**	15205	65.0%	15719	67.4%	13408	82.4%	13630	83.8%
**DE Donor and IVF and DE NT and IVF**	2359	10.1%	779	3.3%	4089	25.2%	4531	27.9%
**ON-Memory** (down-regulated in IVF vs Donor, up-regulated in NT vs IVF)	640	2.7%	307	1.3%	1169	7.2%	1317	8.1%
**ON-Memory(3FC)** (down-regulated in IVF vs Donor, >3-fold up-regulated in NT vs IVF)	231	1%	140	0.6%	194	1.2%	30	0.2%
**OFF-Memory** (up-regulated in IVF vs Donor, down-regulated in NT vs IVF)	796	3.4%	266	1.1%	1139	7.0%	1404	8.6%
**OFF-Memory(3FC)** (up-regulated in IVF vs Donor, >3-fold down-regulated in NT vs IVF)	183	0.8%	63	0.3%	72	0.4%	37.0	0.2%
**Reprogrammed** (up- or down-regulated in IVF vs Donor, and not DE in IVF vs NT)	10825	46.4%	11625	49.9%	9319	57.3%	9787	60.2%
**Reprogrammed-down** (down-regulated in IVF vs Donor, and not DE in IVF vs NT)	5666	24.3%	6024	25.8%	4560	28.0%	4645	28.6%
**Reprogrammed-up** (up-regulated in IVF vs Donor, and not DE in IVF vs NT)	5159	22.1%	5601	24.0%	4160	25.6%	3883	23.9%

ID, identified; Donor, Endoderm donor cells; NT, ectoderm of nuclear transfer embryo, IVF, Ectoderm of *in vitro* fertilized embryo; DE, differentially expressed; CPM, counts per million; FDR, false discovery rate; FC, fold change; For the filters applied, see Material and Methods.

Table S4; Related to STAR Methods; Primer table.

**Gene expression analysis (Fig.S1 and S2)**	
Name	Sequence
a2m-Fwd	GACGGTGCGCAAATATTTCC
a2m-Rev	AGCGTTCCCATCAGCATCTG
gata6-Fwd	CGATGCGTTCCCCTTCTG
gata6-Rev	ACAAGTCCACAGTTTTCATCAACAG
sox17β -Fwd	CGTCCTGGGCTGGAGATGT
sox17β-Rev	TCTCCTCTGGATTTGGCAGAA
foxA4-Fwd	TGTCCCCTCCTGGTGGAA
foxA4-Rev	TGGTGCCTCCCTGGAAGAC
darmin-Fwd	CCCCTGTGTCAGCTTGCAT
darmin-Rev	TGGGTGAAAATGAAACAGATTTGT
H4-Fwd	GACGCTGTCACCTACACCGAG
H4-Rev	CGCCGAAGCCGTAGAGAGTG
**ChIP analysis (Fig.S2)**	
gata6-Fwd-	CAAGTACTGGGAGCTGTACCACAA
gata6-Rev	AATTATGCTGCTAAGGGACAGACA
gata6-Fwd	CGGTGGTTGCGCGATATAG
gata6-Rev	CCAAGGAGCCATTGTGCAT
sox17β-Fwd	TCCCGCATCGCTCTTCAG
sox17β-Rev	TGGGCCGAACCCATGAC
sox17β-Fwd	GGGATGTTTGCACTTGGAAAG
sox17β-Rev	AGGAAGAAGCAGGTGAAGAGGAT
foxA4-Fwd	TGGACTCCAGAACATGCTAAATAGA
foxA4-Rev	TTGGTACATGGTATTCCAGTCCAT
foxA4-Fwd	TGTCCCCTCCTGGTGGAA
foxA4-Rev	TGGTGCCTCCCTGGAAGAC
darmin-Fwd	CCCCATGTGCCCCTAGCT
darmin-Rev	CAGTAGTAGCGCTTTTGAAGCAAA
darmin-Fwd	CAGTTGCCCCTTGCTCCAT
darmin-Rev	TGTCACAGACACACCGTGGTT

Fwd, Forward primer; Rev, reverse primer.
