# Supplementary material for: H3K4 Methylation-Dependent Memory of Somatic Cell Identity Inhibits Reprogramming and Development of Nuclear Transfer Embryos
Source: Cell Stem Cell. 2017 Jul 6;21(1):135–143.e6. doi: 10.1016/j.stem.2017.03.003 (PMC5505866; doi:10.1016/j.stem.2017.03.003)
Supplement: Document S2. Article plus Supplemental Information [file mmc2.pdf]

Short Article

Cell Stem Cell

H3K4 Methylation-Dependent Memory of Somatic Cell Identity Inhibits Reprogramming and Development of Nuclear Transfer Embryos

Graphical Abstract

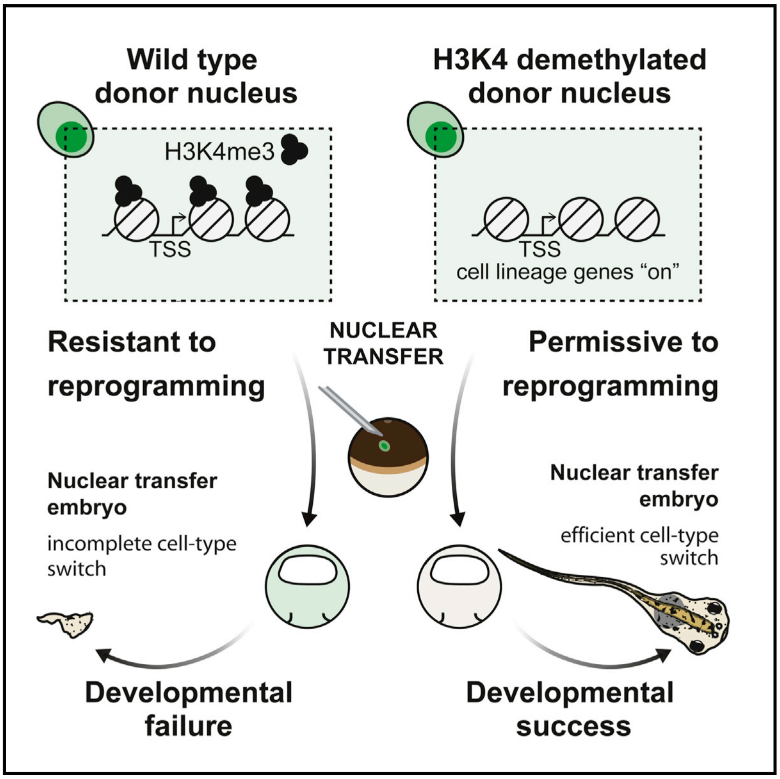

Authors

Eva Hörmanseder, Angela Simeone, George E. Allen, Charles R. Bradshaw, Magdalena Figlmüller, John Gurdon, Jerome Jullien

Correspondence

e.hoermanseder@gurdon.cam.ac.uk (E.H.),
j.jullien@gurdon.cam.ac.uk (J.J.)

In Brief

Hörmanseder et al. find that persistent memories of transcriptional activity in donor cell identity genes present a barrier to cell-fate reprogramming following nuclear transfer. They show that reducing H3K4 methylation in donor cells reduces transcriptional memory and improves the development of embryos derived by nuclear transfer.

Highlights

- Nuclear transfer embryos retain the memory of a past state of active transcription (ON-memory)
- ON-memory genes are enriched for H3K4 methylation in somatic donor nuclei
- H3K4 demethylation improves transcriptional reprogramming
- Removing H3K4 methylation enhances the development of nuclear transfer embryos

Hörmanseder et al., 2017, Cell Stem Cell *21*, 135–143
July 6, 2017 © 2017 The Authors. Published by Elsevier Inc.
http://dx.doi.org/10.1016/j.stem.2017.03.003

H3K4 Methylation-Dependent Memory of Somatic Cell Identity Inhibits Reprogramming and Development of Nuclear Transfer Embryos

Eva Hörmanseder,[1,3,*] Angela Simeone,[1] George E. Allen,[1] Charles R. Bradshaw,[1] Magdalena Figlmüller,[1] John Gurdon,[1,2] and Jerome Jullien[1,*]
[1]Wellcome Trust/Cancer Research UK Gurdon Institute
[2]Department of Zoology
University of Cambridge, Cambridge CB2 1QN, UK
[3]Lead Contact
*Correspondence: e.hoermanseder@gurdon.cam.ac.uk (E.H.), j.jullien@gurdon.cam.ac.uk (J.J.)
http://dx.doi.org/10.1016/j.stem.2017.03.003

SUMMARY

Vertebrate eggs can induce the nuclear reprogramming of somatic cells to enable production of cloned animals. Nuclear reprogramming is relatively inefficient, and the development of the resultant embryos is frequently compromised, in part due to the inappropriate expression of genes previously active in the donor nucleus. Here, we identify H3K4 methylation as a major epigenetic roadblock that limits transcriptional reprogramming and efficient nuclear transfer (NT). Widespread expression of donor-cell-specific genes was observed in inappropriate cell types in NT embryos, limiting their developmental capacity. The expression of these genes in reprogrammed embryos arises from epigenetic memories of a previously active transcriptional state in donor cells that is characterized by high H3K4 methylation. Reducing H3K4 methylation had little effect on gene expression in donor cells, but it substantially improved transcriptional reprogramming and development of NT embryos. These results show that H3K4 methylation imposes a barrier to efficient nuclear reprogramming and suggest approaches for improving reprogramming strategies.

INTRODUCTION

During development, cells lose their pluripotent status and acquire a stable cell identity, which only rarely, if ever, changes to another kind. Yet, somatic cells can be reprogrammed to another cell fate by nuclear transfer (NT) to eggs (Gurdon, 1960), by the expression of a combination of transcription factors (Takahashi and Yamanaka, 2006) or by cell-cell fusion (Blau et al., 1983). In these reprogramming procedures, the gene-expression pattern and epigenetic state characteristic of one differentiated cell identity is erased and the gene expression pattern specific to another cell type is established.

However, the efficiency of complete reprogramming via NT is low, as less than 10% of NT embryos generated from differentiated cells reach adulthood (Gurdon, 1960; Meissner and Jaenisch, 2006). This led to the hypothesis that differentiated cells acquire a resistance to reprogramming procedures, which during normal development, helps to stabilize their cell fate. Due to this resistance, eggs cannot fully reprogram the incoming somatic nuclei, so that embryos with aberrant gene expression patterns arise and normal embryonic development is not supported (Gao et al., 2003; Hirasawa et al., 2013; Ng and Gurdon, 2005). So far, it has been shown that a failure in reactivating genes, e.g., the pluripotency gene Oct4, during nuclear reprogramming is indicative of a poor developmental outcome of NT embryos (Boiani et al., 2002). Furthermore, epigenetic modifications inhibiting the re-activation of genes during the reprogramming procedure have been investigated and their removal has been utilized to improve reprogramming efficiency and to increase the viability of NT embryos (Blelloch et al., 2006; Chung et al., 2015; Enright et al., 2003; Kishigami et al., 2006; Liu et al., 2016; Matoba et al., 2014). However, the expression of donor cell-type-specific genes in the wrong cell type of NT embryos could also lead to a severe disruption of normal gene expression patterns resulting in developmental defects and embryonic lethality. Indeed, the existence of such an active transcription state memory has been suggested in NT and induced pluripotent stem cell (iPSC) experiments (Polo et al., 2010; Kim et al., 2011; Ng and Gurdon, 2005). Currently, however, the extent and functional importance of persistent donor-cell-type-specific gene expression in resistance to reprogramming is not known. Furthermore, the epigenetic mechanisms that confer memory of an active state of gene expression and that maintain the differentiated state of cells during nuclear reprogramming and embryonic development remain elusive.

Here we show that in *Xenopus* and human NT embryos, memory of an active transcriptional state (ON-memory) is a phenomenon as widespread as the memory of an inactive transcriptional state. ON-memory genes are associated with increased levels of the active histone mark H3K4me3 when compared to properly reprogrammed genes in *Xenopus* and human somatic donor cells. Importantly, while a reduction in H3K4 methylation levels has little effect on gene expression in the donor cells, it significantly improves transcriptional reprogramming and enhances

This is an open access article under the CC BY license (http://creativecommons.org/licenses/by/4.0/).

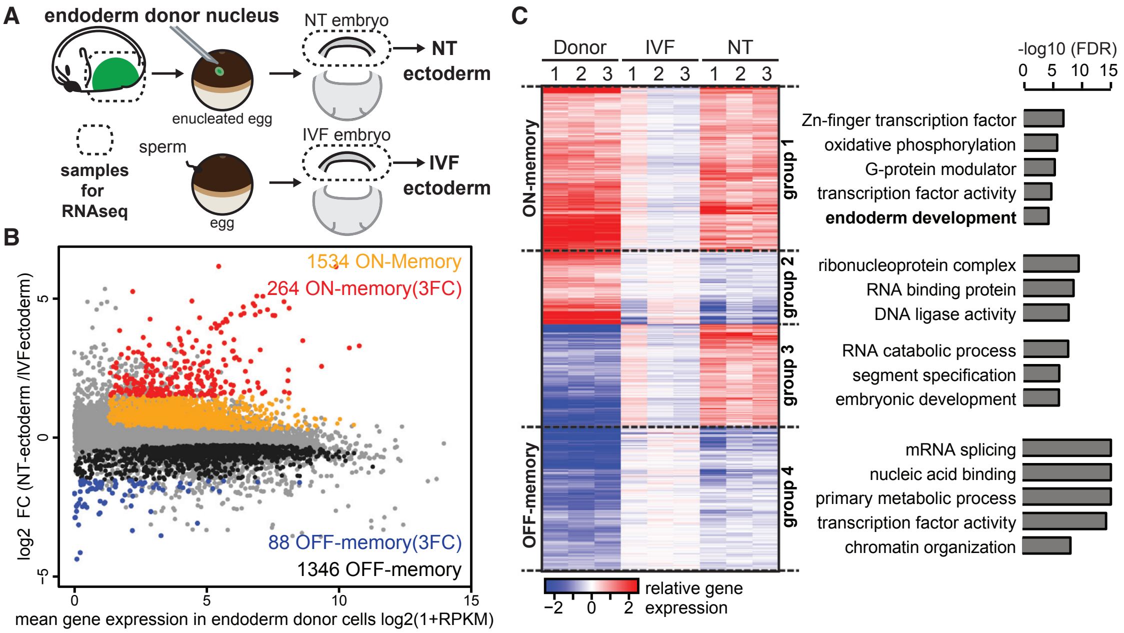

Figure 1. Donor Cell-Type-Specific Genes Are Expressed in the Wrong Cell Type of NT Embryos

(A) Design of NT experiments.

(B) MA plot comparing gene expression between ectoderm of NT and IVF embryos. The average log2 FC in expression of transcripts in NT embryos over IVF embryos is plotted on the y axis, and the mean log2 (1+RPKM) gene expression in the donor endoderm cells is plotted on the x axis. Gray, all transcripts; orange, ON-memory genes; black, OFF-memory genes; red, ON-memory(3FC) genes; blue, OFF-memory(3FC) genes.

(C) Heatmap showing 4,504 differentially expressed transcripts obtained by pairwise comparison between donor endoderm cells and IVF and NT ectoderm cells. Rows are log2 FC in expression over mean expression levels in IVF. Hierarchical clustering of rows classified those genes into four groups. Gene ontology analysis revealed that ON-memory genes are enriched for genes important for endoderm development; FDR, false discovery rate; FC, fold change.

See also Figures S1 and S4 and Tables S1 and S2.

the developmental potential of the resultant NT embryos in *Xenopus*. Our study thus identifies H3K4 methylation as a critical epigenetic barrier in NT-mediated reprogramming and implicates its role as stabilization mechanism of cell differentiation.

RESULTS

Identification of Reprogramming Resistant ON-Memory Genes Expressed in the Wrong Cell Type of NT Embryos

The low success rate of current cloning strategies was suggested to be partly due to the persistence of a donor-cell-type-specific gene expression pattern in NT embryos, which could hinder the generation of new cell types (Firas et al., 2014; Liu et al., 2016; Matoba et al., 2014). As a first step to test this hypothesis, we evaluated the extent of memory gene expression in *Xenopus* NT embryos on a transcriptome-wide level.

For this purpose, the nucleus of a neurula-stage endoderm cell was transplanted to an enucleated egg to obtain NT embryos and as a control for normal gene expression, in vitro fertilized (IVF) embryos were generated (Figure 1A). Properly cleaved embryos were collected at the gastrula stage, a time point where ectoderm and endoderm identity is established and before any developmental defects can be observed in these NT embryos. Endoderm donor cells as well as ectoderm cells of single NT and IVF embryos were then subjected to RNA sequencing (RNA-seq) analysis in biological triplicate (Figures 1A and S1A–S1F; Tables S1 and S2). To test the extent of memory and reprogramming in the newly generated cell type, we addressed which transcripts differ between endoderm donor cells and ectoderm cells of IVF embryos. When the expression of these genes also differs between NT- and IVF- ectoderm cells, we consider them to be examples of donor cell memory (Figure S1A). If they are expressed at similar levels in NT and IVF, we consider them as reprogrammed (Figure S1B). Of all 24,215 identified transcripts (Figure 1B, in gray), a large number (17,587; Table S2) was differentially expressed between endoderm donor cells and ectoderm cells of IVF embryos. 13,083 of these genes were reprogrammed as they were expressed at similar levels in the ectoderm of NT and control IVF embryos (Table S2). In contrast, 4,504 genes were resistant to reprogramming as they were differentially expressed between ectoderm cells of NT and control IVF embryos (Figures 1B and 1C). This gene set included 1,534 ON-memory genes-these are genes that were expressed in donor endoderm cells and continued to be significantly (false discovery rate [FDR] $\leq$ 0.05) upregulated in NT ectoderm cells when compared to IVF ectoderm cells (Figures 1B and 1C, group 1). Another 1,346 of the same gene set are described as OFF-memory genes, because their transcripts were expressed at significantly (FDR $\leq$ 0.05) lower levels in ectoderm cells of NT embryos when compared to IVF controls (Figure 1B and 1C, group 4). The remaining 1,624 genes were either too much down- or upregulated in the ectoderm of NT embryos when compared to the IVF controls (Figure 1C, group 2 and group 3, respectively). We therefore see that a total of 2,880 ON-memory and OFF-memory

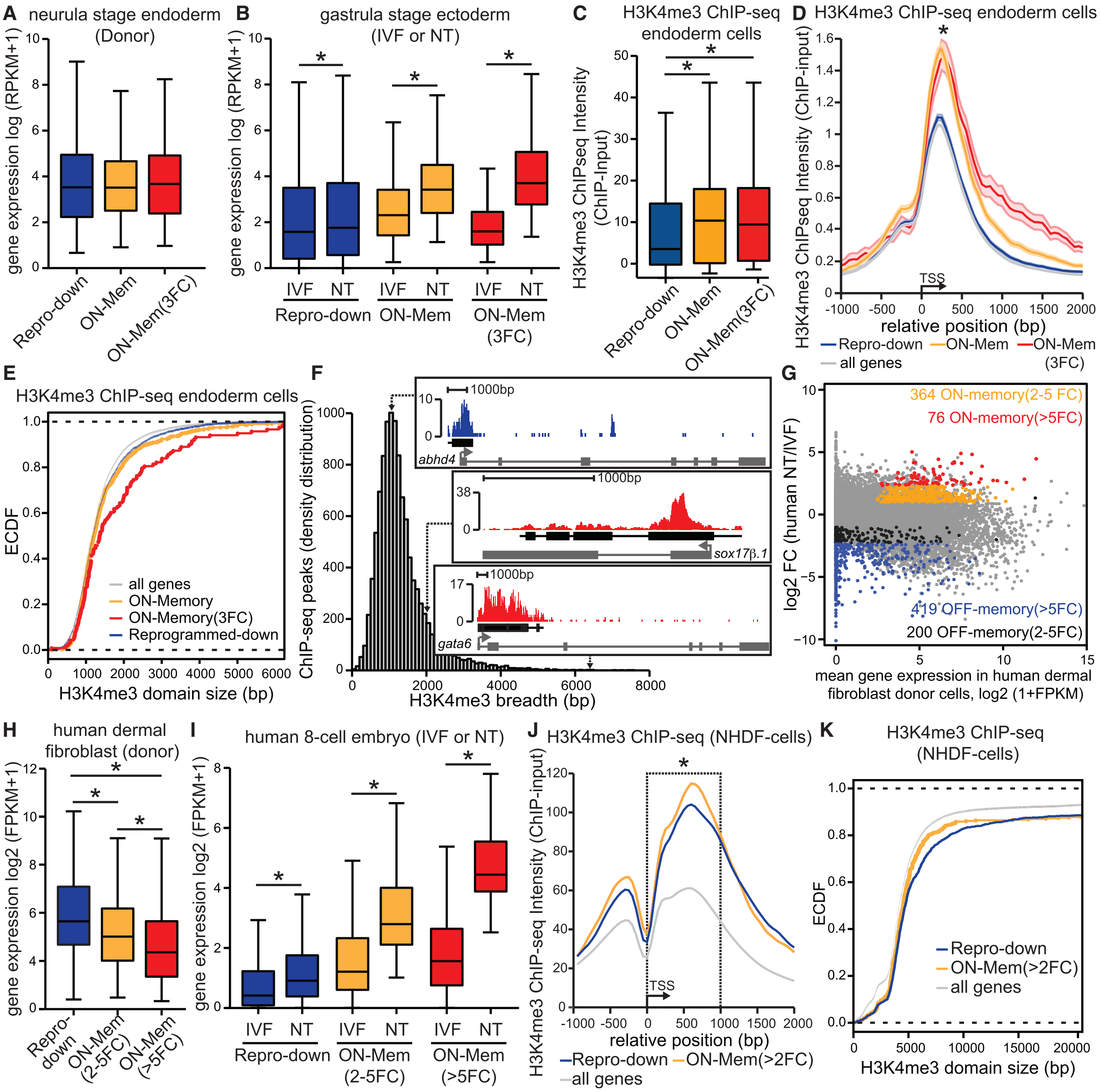

Figure 2. ON-Memory Genes Are Enriched for H3K4me3 in *Xenopus* and Human Donor Cells

(A) Reprogrammed-down, ON-memory, and ON-memory(3FC) genes have similar expression levels in donor-endoderm cells (p values > 0.4, Mann-Whitney test).

(B) ON-memory-genes are upregulated in NT cells when compared to IVF ectoderm cells. Boxplot comparing mean expression levels of reprogrammed-down (*p value = 1.024×10^{-7}), ON-memory (*p value < 2.2×10^{-16}), and ON-memory(3FC)-genes (*p value < 2.2×10^{-16}); Mann-Whitney test.

(C–F) H3K4me3 ChIP-seq data generated from neurula-stage endoderm cells. Read counts are normalized by input and total mapped reads. (C) ON-memory-genes are enriched for H3K4me3 in donor-endoderm cells. Boxplot comparing mean H3K4me3 ChIP-seq intensities in a 4-kb window centered on the TSS (*p value < 0.001, KS test). (D) TSS metaplots of H3K4me3 ChIP-seq intensity in endoderm cells. ON-memory(3FC) and ON-memory ChIP-seq intensities are higher when compared to reprogrammed-down genes (p value = 0.07 and *p value = 0.001, respectively KS test). (E) ON-memory(3FC)-genes show increased H3K4me3 breadth when compared to reprogrammed-down genes (p value = 0.0002, KS test). Empirical cumulative distribution function (ECDF) comparing H3K4me3 domain size spanning the TSS. (F) Breadth distribution of H3K4me3 ChIP-seq peaks. Inserts are examples of H3K4me3 regions of a reprogrammed-down gene (*abhd4*) and ON-memory genes *sox17β.1* and *gata6*(NM_001087983.1).

(G) MA plot comparing gene expression between human NT and IVF embryos. The average log2 FC in expression of transcripts in NT embryo over IVF embryos (n = 1; pool of five NT and of five IVF 8-cell embryos) is plotted on the y axis, and the mean log2 (1+FPKM) gene expression in the endoderm donor cells (1 sample of the donor dermal fibroblast cells, DFB-8) is plotted on the x axis. Gray, all identified transcripts; orange, ON-memory(2-5FC); black, OFF-memory(2-5FC); red, ON-memory(> 5FC) genes; blue, OFF-memory(> 5FC) genes.

(legend continued on next page)

genes are not reprogrammed by NT to eggs in *Xenopus*, and instead remember their donor cell expression pattern. Furthermore, this result suggests that NT embryos show endoderm ON-memory gene expression to the same extent as OFF-memory gene expression in the newly generated ectoderm cell type.

To obtain insight into biological processes associated with the inappropriately reprogrammed genes, we performed gene ontology analysis. This revealed that as a whole, reprogramming resistant genes are enriched for genes involved in development, transcriptional activity, and metabolic processes (Figure 1C). Importantly, we observed that the ON-memory gene set was enriched for genes implicated in endoderm development (Figure 1C, group 1). Furthermore, we found that master regulators of endoderm specification in *Xenopus*, such as *sox17* and *gata6*, were among the ON-memory genes showing the highest upregulation in NT embryos when compared to IVF (Figure S1C). Hence, these results point toward retention of endoderm donor cell identity in the ectoderm cells of the NT embryos.

Next, we investigated the mechanism by which the ON-memory gene transcripts are accumulated in the ectoderm cells of the NT embryos. In *Xenopus*, there is no transcription for the first 12 cell cycles of embryonic development (Hörmanseder et al., 2013). Consistently, we did not observe gene expression of the ON-memory genes *sox17β*, *gata6*, and *a2m (endodermin)* at stage 7, prior to zygotic genome activation (ZGA; Figures S1G and S1H). This indicates that there was no carry-over of transcripts for these genes during NT, and that transcripts detected here were newly synthetized after ZGA. We therefore conclude that the memory of an active state of gene transcription of the donor nucleus was transmitted to its mitotic progeny during early embryonic cell divisions in the absence of the conditions that induced that state, and independently of ongoing gene transcription. It implies that the memory of the donor cell gene expression pattern observed in NT embryos is stabilized by epigenetic mechanisms.

ON-Memory Genes Are Enriched for H3K4me3 in Endoderm Donor Nuclei in *Xenopus*

We then investigated which epigenetic feature of the donor nuclei could account for the fact that ON-memory genes resist the reprogramming process and commence expression in the wrong cell type of NT embryos. Actively transcribed genes are characterized by the presence of methylated lysine 4 on histone H3 (H3K4me3) (Santos-Rosa et al., 2002). We hypothesized that accumulation of H3K4me3 following transcription of endoderm genes in donor cells could confer ON-memory gene-expression after NT.

Our transcriptome analysis identified reprogrammed-down genes that were active in endoderm donor cells (Figure 2A) but are reprogrammed and downregulated to IVF levels in the ectoderm of NT embryos (Figure 2B). ON-memory genes were initially expressed in endoderm donor cells at similar levels to reprogrammed-down genes (Figure 2A). However, they remained significantly upregulated in ectoderm cells of the NT embryo when compared to IVF (ON-memory; Figure 2B). Within the ON-memory group, a subset were especially resistant to reprogramming as they were more than 3-fold overexpressed (ON-memory(3FC); Figure 2B). Using these three sets of genes we tested if differences in H3K4me3 features could explain resistance to transcriptional reprogramming. We performed H3K4me3 chromatin immunoprecipitation sequencing (ChIP-seq) analysis on neurula-stage endoderm donor cells in biological duplicate (Figures 2C–2F and S2A–S2D). The intensity of the H3K4me3 ChIP-seq signal around the transcriptional start site (TSS) was significantly higher in ON-memory genes when compared to reprogrammed-down genes (Figures 2C and 2D). Previous studies suggested that broad H3K4me3 domains are linked to cell identity and transcriptional consistency (Benayoun et al., 2014), and since endoderm ON-memory genes are also enriched for endoderm lineage genes and maintain their transcriptionally active state even after the nuclear reprogramming procedure, we tested if ON-memory genes showed high H3K4me3 breadth. Indeed, when comparing the empirical cumulative distribution of H3K4me3 domain size spanning the TSS, ON-memory(3FC) genes showed significantly broader H3K4me3 domains than reprogrammed-down genes (Figure 2E). For example, the ON-memory genes *gata6* and *sox17β* are marked by broader domains than the reprogrammed-down gene *abhd4* (Figure 2F).

These results suggest that ON-memory genes are enriched for H3K4me3, as they show higher ChIP-seq intensity and broader domains of this mark when compared to reprogrammed-down genes in the endoderm donor cells. Increased H3K4me3 levels and breadth could act together as barrier to cell-fate changes and hence explain why the set of memory-ON genes are resisting the reprogramming process.

The Phenomenon of ON-Memory Is Conserved in Human NT Embryos, and ON-Memory Genes Are Enriched for H3K4me3 in Human Donor Cells

Our observations in *Xenopus* prompted us to investigate if ON-memory gene expression is conserved in human NT embryos and whether ON-memory genes, when compared to reprogrammed-down genes, are also enriched for H3K4me3 levels in

(H) Boxplot comparing mean expression levels of reprogrammed-down, ON-memory(2-5FC), and ON-memory(> 5FC) genes in DFB-8 donor cells (*p values < 0.002, Mann-Whitney test).

(I) ON-memory genes are upregulated in eight-cell NT embryos when compared to IVF embryos. Boxplot comparing mean expression levels of reprogrammed-down, ON-memory(2-5FC), and ON-memory(> 5FC) genes (*p values $< 2.2\times 10^{-16}$) in eight-cell NT and IVF embryos; statistical test: Mann-Whitney test.

(J and K) H3K4me3 ChIP-seq datasets of H3K4me3 in human dermal fibroblast cells (NHDF-cells) were obtained from the ENCODE project (Consortium, 2012).

(J) TSS meta-plots of the average intensity of H3K4me3 modifications in NDHF cells for reprogrammed-down, ON-memory(> 2FC), and all genes of the human genome. ON-memory(> 2FC) ChIP-seq intensities are significantly higher when compared to reprogrammed-down genes (*p value < 0.035, 1 kb window around the TSS, KS test). (K) ECDF comparing H3K4me3 domain size around the TSS of reprogrammed-down, ON-memory(> 2FC), and all genes from the human genome. ON-memory(> 2FC)-genes do not show a significant increase in H3K4me3 breadth when compared to reprogrammed-down genes (p value = 0.85, KS test; ChIP-seq peaks called by MACS2).

Boxplots: middle line in the box indicates the median; box edges indicate 25th/75th percentiles; and whiskers indicate min and max. See also Figures S2 and S4 and Table S2.

the human donor cells. In previous studies (Chung et al., 2015; Matoba et al., 2014), epigenetic marks correlating with ON-memory genes were not addressed. Therefore, we obtained the published RNAseq datasets generated from pools of human eight-cell IVF embryos, as well as from eight-cell NT embryos using human dermal fibroblast (DFB-8) cell nuclei as donors (Chung et al., 2015).

Our transcriptome comparison between the donor DFB cells and IVF and NT embryos identified a set of 76 ON-memory genes that remained more than 5-fold upregulated in NT embryos when compared to IVF control (ON-memory(> 5FC); Figures 2G–2I). A set of 364 genes showed partial inactivation (ON-memory(2-5 FC)) (Figures 2G–2I) and a third set of 508 genes was efficiently downregulated in the NT-embryo when compared to IVF (reprogrammed-down; Figures 2I and 2J).

Therefore, our analysis of human NT embryo RNA-seq data suggests that the phenomenon of ON-memory gene expression is conserved in human NT embryos.

We then investigated the H3K4me3 ChIP-seq intensities of ON-memory and reprogrammed-down genes using publicly available H3K4me3 ChIP-seq datasets for NHDF cells, which are related to the DFB-8 cells used as donors to generate the NT embryos. In agreement with the results obtained for *Xenopus*, ON-memory genes showed increased H3K4me3 intensity around their TSS when compared to reprogrammed-down genes (Figure 2J). However, when comparing H3K4me3 domains breadth of ON-memory genes and reprogrammed-down genes, we could not observe a significant difference (Figure 2K).

These results suggest that also in human, high H3K4me3 levels at the TSS could confer ON-memory gene expression in NT embryos and hence act as barrier to nuclear reprogramming.

H3K4 Demethylation of Donor Nuclei Improves Transcriptional Reprogramming in *Xenopus* NT Embryos

Having established a correlation between ON-memory gene expression and H3K4me3 enrichment, we next asked whether this modification is responsible for resistance to reprogramming.

Hence, one-cell embryos were injected with mRNA encoding the H3K4-specific demethylase Kdm5b^{wt}, or with Kdm5b^{ci}, the catalytic inactive version of the enzyme, and grown to neurula-stage (Figure 3A). Western blot analysis confirmed that Kdm5b^{wt}-expressing embryos showed reduced H3K4me3 levels when compared to uninjected embryos (Figure 3B). Furthermore, H3K4me3 ChIP-RTqPCR analysis verified the reduction in H3K4me3 levels following Kdm5b^{wt} treatment around the TSS and in the gene body of candidate ON-memory genes (Figure S2E). We then used Kdm5b^{wt}- and Kdm5b^{ci}-expressing neurula-stage endoderm cells as donors to generate NT(Kdm5b^{wt}) embryos and NT(Kdm5b^{ci}) embryos, respectively (Figure 3A). As controls, we in vitro fertilized embryos. We collected gastrula stage embryos and subjected them, as well as the endoderm donor cells, to RNA-seq analysis (Figure 3A; Tables S1 and S3).

First, we addressed the effect of H3K4 demethylation on gene expression in donor cells. Interestingly, we identified that only 102 out of 24,758 identified transcripts were differentially expressed between Kdm5b^{WT} and Kdm5b^{ci} treated donor cells (Table S3). Therefore, changes in H3K4 methylation do not result in strong changes of gene expression levels, as reported previously (Clouaire et al., 2012). Second, we addressed if H3K4 methylation is important for stabilizing an active state of gene expression by evaluating if genes lose resistance to reprogramming as well as their ON-memory state following Kdm5b^{wt} treatment of the donor cell. Transcriptome comparison of Kdm5b^{ci} expressing endoderm donor cells, the ectoderm of IVF embryos and NT(Kdm5b^{ci}) embryos identified 1,434 reprogramming resistant genes as they were differentially expressed between ectoderm cells of NT(Kdm5b^{ci}) and IVF embryos (Figure 3C). By comparison, in NT(Kdm5b^{wt}) embryos, the number of reprogramming resistant genes was substantially reduced, as our analysis identified only 573 differentially expressed genes between the ectoderms of NT(Kdm5b^{wt}) embryos and IVF embryos (Figure 3D). Importantly, Kdm5b^{wt} treatment of the donor cells significantly reduced ON-memory gene expression from 231 ON-memory(3FC) genes in NT(Kdm5b^{ci}) embryos (Figure 3C) to 140 ON-memory(3FC) genes in NT(Kdm5b^{wt}) embryos (Figure 3D). While the expression levels of ON-memory genes in the donor tissues was unaffected by the treatment with Kdm5b^{wt} when compared to Kdm5b^{ci}, we observed a significant reduction of average ON-memory gene expression levels in NT(Kdm5b^{wt}) embryos when compared to NT(Kdm5b^{ci}) embryos (Figures 3E and 3F). Hierarchical clustering (Figure 3G), as well as principal component analysis (PCA) (Figures S2F and S2G) of the transcriptome revealed that three out of seven NT(Kdm5b^{wt}) embryos have a gene expression pattern in their ectoderm cells that is more similar to the one of IVF embryos than to the one of control NT(Kdm5b^{ci}) embryos.

This implicates that expression of Kdm5b^{wt} in the donor cell reduces ON-memory gene expression and is able to improve the whole transcriptome of the resultant NT embryos.

Next, we analyzed the expression of selected candidate ON-memory genes (*sox17β*, *gata6*, *foxA4*, *a2m*, and *darmin)* during gastrulation of NT and IVF embryos via qRT-PCR. In donor endoderm cells, ON-memory gene expression was not affected by H3K4 demethylation (Figures S2H–S2L). We observed that ON-memory genes were upregulated in NT(Kdm5b^{ci}) ectoderm cells at all stages of gastrula embryos (Figures S2M–S2Q). While *sox17β*, *gata6* and *foxA4* showed a decrease in gene expression in NT(Kdm5b^{wt}) ectoderm cells at all stages (Figures S2M–S2O), *a2m* and *darmin* were insensitive to Kdm5b^{wt} treatment of the donor cell as they did not show a significant reduction in gene expression in the resultant NT(Kdm5b^{wt}) ectoderm cells when compared to NT(Kdm5b^{ci}) ectoderm cells (Figures S2P–S2Q). We propose that this is due to an additional, unknown epigenetic barrier other than H3K4me3, as we could observe that H3K4me3 levels were reduced at the TSS and at the gene body of *darmin* to a similar extent as of the other, Kdm5b^{wt} sensitive ON-memory genes (Figure S2E). These results corroborate that Kdm5b^{wt} treatment can reduce ON-memory gene expression in the resultant NT embryos throughout gastrulation.

Finally, we confirmed that the observed reduction in ON-memory gene expression following Kdm5b^{wt} expression is indeed due to the demethylation of H3K4. We reduced H3K4me3 levels in the donor cells by expressing a dominant-negative version of histone H3.3 (H3.3^{K4M}) that binds and inhibits the SET domain of H3K4-specific methyltransferases (Lewis et al., 2013). Transcriptome comparison between donor cells, the ectoderm cells of IVF embryos and of the NT(H3.3^{wt}) embryos or NT(H3.3 ^{K4M})

CellPress

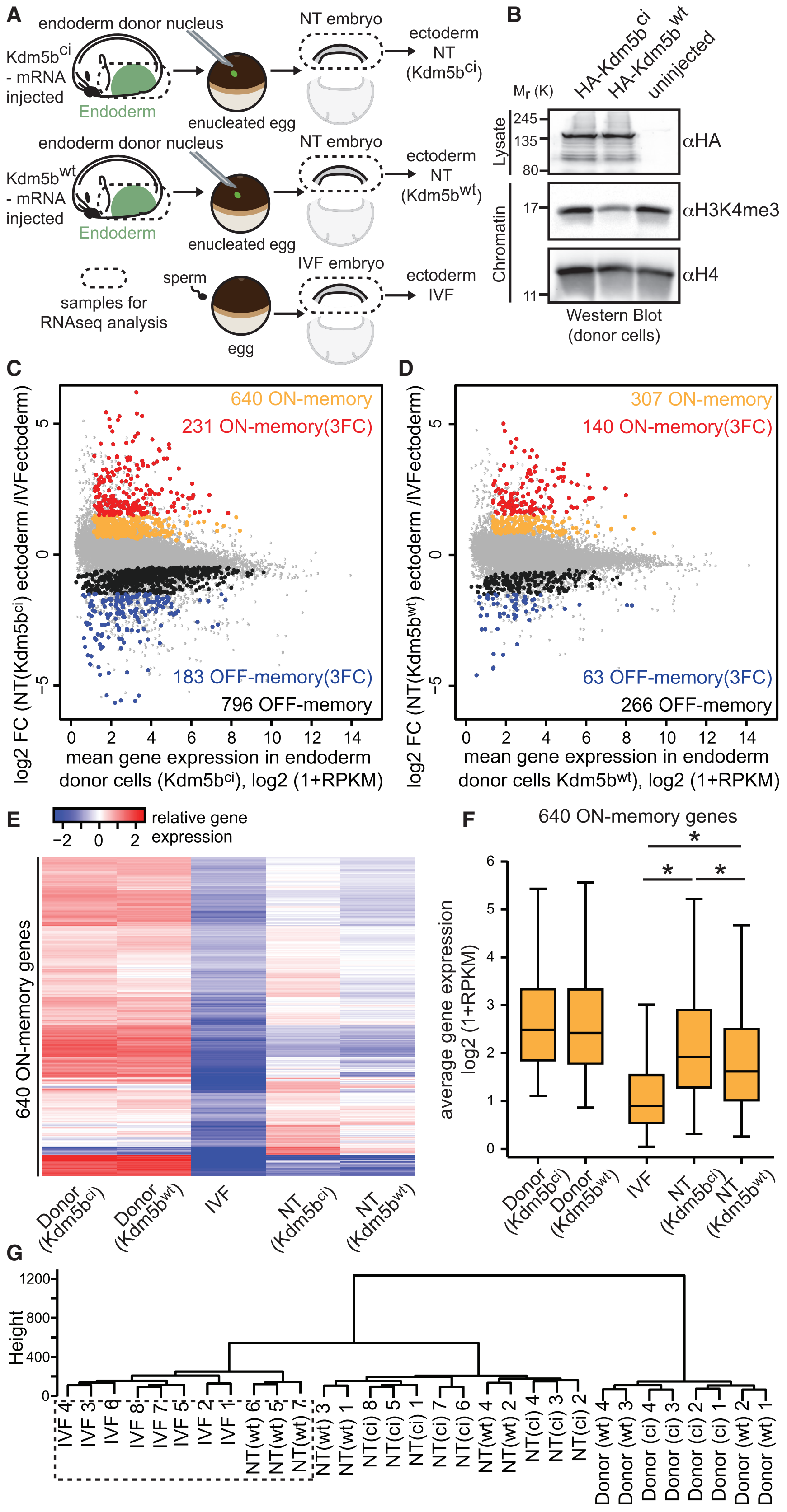

Figure 3. Kdm5b Expression in the Donor Nuclei Reduces H3K4me3 Levels and Improves Reprogramming in NT Embryos

(A) Design of NT experiments.

(B) Western blot analysis indicating that Kdm5b^{WT} expression reduces H3K4me3 by ≈70% in neurula-stage embryos, when compared to uninjected ones.

(C and D) Kdm5b^{wt} expression in the donor cells reduces the number of misregulated genes in NT embryos when compared to IVF embryos. MA plot comparing gene expression between NT(Kdm5b^{ci}) and IVF ectoderm cells (C) or NT(Kdm5b^{wt}) and IVF ectoderm cells (D). Average log2 FC in expression of transcripts in NT over IVF ectoderm cells is plotted on the y axis, and the mean log2 (1+RPKM) gene expression in the donor-endoderm cells is plotted on the x axis. Gray, all transcripts; orange, ON-memory genes; black, OFF-memory genes; red, ON-memory(3FC)genes; blue, OFF-memory(3FC) genes.

(E) ON-memory gene expression is reduced in NT(Kdm5b^{wt}) embryos. Heatmap showing the expression of ON-memory genes identified in NT(Kdm5b^{ci}) embryos in donor-endoderm cells in IVF, NT(Kdm5b^{ci}), and NT(Kdm5b^{wt}) ectoderm cells. Rows represent log2 FC in expression of the indicated samples over mean pooled expression levels of all samples. Rows were sorted by hierarchical clustering.

(F) Boxplots comparing mean expression levels of ON-memory transcripts in donor-endoderm cells and IVF, NT(Kdm5b^{ci}), and NT(Kdm5b^{wt}) ectoderm cells. (*p values < 0.0004, Mann-Whitney test.)

(G) Hierarchical transcriptome clustering analysis of filtered and normalized RNAseq data of single ectoderm tissues of IVF, NT(Kdm5b^{wt}), and NT(Kdm5b^{ci}) embryos, as well as donor endoderm cells.

Boxplots: middle line in the box indicates the median; box edges indicate 25th/75th percentiles; and whiskers indicate min and max. See also Figures S2–S4 and Tables S1 and S3.

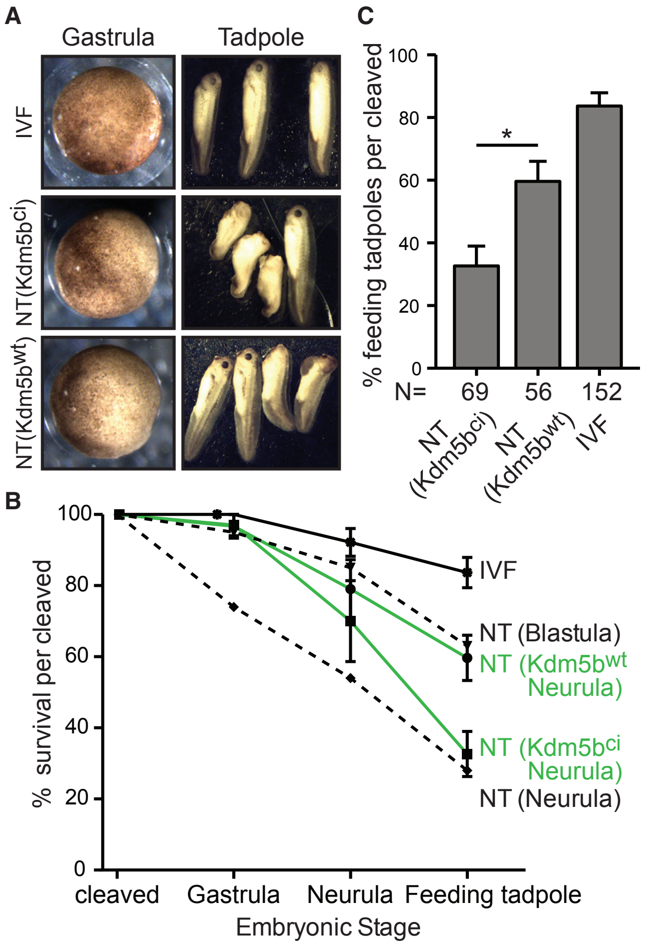

Figure 4. H3K4 Demethylation in Donor Nuclei Improves the Development of NT Embryos

(A) IVF, NT(Kdm5b^{ci}), and NT(Kdm5b^{wt}) embryos at the gastrula and tadpole stages.

(B) The development of IVF, NT(Kdm5b^{ci}), and NT(Kdm5b^{ci}) gastrula embryos (stage 10.5) was followed until feeding tadpole stage (green lines and black solid line, respectively). Black dashed lines indicate the developmental potential of NT embryos generated from uninjected blastula stage or neurula stage endoderm nuclei (data from Gurdon, 1960). y axis is the percentage of gastrula embryos reaching the indicated stages.

(C) Kdm5b^{WT} expression in the donor cell improves the development of NT embryos to the feeding tadpole stage. Bar graph showing the percentage of cleaved gastrula embryos reaching the feeding tadpole stage (*p value = 0.0007, paired t test, one-tailed). Data are presented as mean ± SEM.

See also Figure S4 and Table S1.

embryos corroborated the finding that a reduction of H3K4 methylation decreases ON-memory gene expression in NT embryos (Figure S3; Table S3).

Together, our data show that a H3K4 demethylation of donor nuclei not only improves the reprogramming of ON-memory genes but can also restore the global transcriptome of NT embryos.

H3K4 Demethylation in Donor Nuclei Improves the Development of NT Embryos

Finally, we investigated whether H3K4 demethylation in donor cells and the associated reduction in ON-memory gene expression in the resultant NT embryos are able to improve their survival.

We generated NT embryos from Kdm5b^{wt}- and Kdm5b^{ci}-treated donor cells, and while they developed to properly cleaved early gastrula embryos at a similar rate (Figures 4A and 4B; Table S1), NT(Kdm5b^{wt}) embryos showed fewer morphological abnormalities when compared to NT(Kdm5b^{ci}) embryos (Figures 4A and 4B; Table S1) as development proceeded. Notably, NT(Kdm5b^{wt}) embryos reached the feeding tadpole stage and beyond at a significantly higher rate than NT(Kdm5b^{ci}) embryos (Figure 4C). The developmental potential of NT(Kdm5b^{ci}) embryos was consistent with previous results using uninjected endoderm cells as donors for NT (Gurdon, 1960), as 30% of cleaved NT(Kdm5b^{ci}) embryos reached the feeding tadpole stage (Figures 4B and 4C). Instead, the developmental outcome of NT(Kdm5b^{wt}) embryos was comparable to the one of NT embryos generated from undifferentiated blastula cells (Gurdon, 1960), as 60% of all cleaved embryos reached the feeding tadpole stage (Figures 4B and 4C).

These results show that H3K4 methylation acts as a barrier to nuclear reprogramming, and that its removal significantly improves the developmental potential of NT embryos.

DISCUSSION

Our study identifies H3K4 methylation as an epigenetic barrier to nuclear reprogramming and suggests it as a safeguarding mechanism for cellular identity. Challenging the stability of cell identity through NT reveals epigenetic mechanisms that inhibit the activation of genes supporting alternative cell fates (Chung et al., 2015; Liu et al., 2016; Matoba et al., 2014). Here, we describe an epigenetic layer that prevents the inactivation of genes during nuclear reprogramming and ensures the stable expression of genes characteristic of an established cell identity. We observe that H3K4 methylation imposes memory of an active transcriptional state and that its suppression results in improved transcriptional reprogramming and an enhancement of the developmental outcome of *Xenopus* NT embryos. Therefore, our study shows that interfering with an epigenetic barrier and the associated ON-memory can improve the generation of new cell types by reprogramming via NT.

By transplanting differentiated nuclei to eggs, we uncover a function of H3K4 methylation in epigenetic memory of cell fate that extends beyond ensuring transcriptional consistency and maintaining ongoing gene transcription (Benayoun et al., 2014). Indeed, reduction of H3K4 methylation by either demethylase or histone mutant expression has very little effect on endodermal cell transcription in fertilized embryos. However, the role of H3K4me3 in stabilizing a transcriptional program becomes most evident when the endoderm cell chromatin undergoes reprogramming by the egg: The early phase of frog embryogenesis encompasses a period of intense cell division in the absence of transcription. After ZGA, when cells are again permissive for transcription, H3K4me3 can induce endoderm ON-memory gene expression in ectoderm cells of cloned embryos. Hence we can differentiate in our experimental system the function of H3K4me3 in simply maintaining ongoing transcription, as observed in pluripotent cells (Muramoto et al., 2010), from its function as an epigenetic memory factor of somatic cell identity.

The stabilization of an active transcriptional state correlates with increased intensity and breadth of H3K4 methylation around these genes, as for example around the endoderm lineage genes *sox17β* and *gata6*. During DNA replication, the modified nucleosomes are locally redistributed between the two daughter strands (Probst et al., 2009) and broad H3K4me3 domains of ON-memory genes could ensure that the mark on these key lineage genes is faithfully propagated to the mitotic progeny, even when the chromatin is challenged by the egg's reprogramming factors and in the absence of gene transcription. Our results underline the importance of H3K4me3 as a safeguarding mechanism of cell identity.

ON-memory gene expression is conserved in *Xenopus* and human NT embryos and correlates with increased H3K4me3 levels in the somatic donor cell nuclei. It is likely that also in mammals, H3K4 methylation imposes a barrier to nuclear reprogramming and its removal enhances the efficiency of cloning. Interestingly, recent studies in mouse NT embryos suggest that a failure in reactivating Kdm5b expression during reprogramming correlates with a poor developmental outcome (Liu et al., 2016), which would support that NT embryos with reduced Kdm5b levels might not be able to efficiently erase H3K4 methylation mediated ON-memory. However, studies also show that Kdm5b knock down in IVF embryos results in aberrant major ZGA (Dahl et al., 2016) and embryonic lethality. It is currently unknown if the developmental failure of mouse NT embryos is due to the normal requirement of Kdm5b for ZGA or if it is due to persistent ON-memory gene expression. In our work, we erase H3K4 methylation marks in the donor cells, and leave the H3K4me3 demethylation activities of the NT embryo unperturbed, and hence are able to show that ON-memory indeed acts as barrier to nuclear reprogramming. We propose that both, ON-memory gene expression due to persistent active marks and as well as a lack in Kdm5b activities, which are important for ZGA and development (Dahl et al., 2016), contribute to embryonic lethality in mouse NT embryos.

Once the essential epigenetic barriers conferring ON- and OFF-memory are identified, they can be targeted to improve reprogramming efficiencies and allow the generation of high quality stem cells suitable for cell replacement therapies.

STAR★METHODS

Detailed methods are provided in the online version of this paper and include the following:

- KEY RESOURCES TABLE
- CONTACT FOR REAGENT AND RESOURCE SHARING
- EXPERIMENTAL MODEL AND SUBJECT DETAILS
- METHOD DETAILS
  - mRNA production
  - mRNA injection into one-cell embryos
  - Donor cell preparation
  - Nuclear transfer and embryo culture
  - RNA extraction
  - cDNA sequencing library
  - cDNA synthesis and RTqPCR analysis
  - Western Blotting
  - Chromatin Immunoprecipitation (ChiP)
  - ChIP-seq library preparation
  - ChIP-RTqPCR
  - Experimental design
- QUANTIFICATION AND STATISTICAL ANALYSIS
  - *Xenopus* transcriptome and sequencing data
  - *Xenopus* differential gene expression
  - Human differential gene expression
  - DE data filter-strategy
  - Heatmaps and plots for gene expression
  - qPCR analysis
  - Principal component analysis and hierarchical transcriptome clustering
  - ChIP-seq data analysis
  - Methylated histone regions
  - Developmental outcome
- DATA AVAILABILITY

SUPPLEMENTAL INFORMATION

Supplemental Information includes four figures and four tables and can be found with this article online at http://dx.doi.org/10.1016/j.stem.2017.03.003.

AUTHOR CONTRIBUTIONS

Conceptualization: E.H.; Methodology: E.H. and J.J.; Software: A.S., G.E.A., and C.R.B.; Formal Analysis: E.H., A.S., and G.E.A.; Investigation: E.H.; Validation: E.H., M.F., and J.J.; Data Curation: C.R.B., A.S., and G.E.A.; Writing – Original Draft and Visualization: E.H.; Writing – Review and Editing: E.H., J.J., and J.G.; Supervision and Funding acquisition: E.H., J.J., and J.G. J.J. and J.G. contributed equally.

ACKNOWLEDGMENTS

This work was funded by the Molecular Research Council (MR/P000479/1), the Wellcome Trust (101050/Z/13/Z and 092096/Z/10/Z), and Cancer Research UK (C6946/A14492). E.H. was a recipient of a long-term fellowship from the European Molecular Biology Organization (EMBO) and Isaac Newton Trust funding. The authors are grateful to Dave Simpson, members of the Gurdon lab and Institute, Julie Ahringer and her lab, and Thomas U. Mayer for support and critically reading the manuscript.

Received: August 23, 2016
Revised: December 5, 2016
Accepted: March 7, 2017
Published: March 30, 2017

REFERENCES

Alexa, A., Rahnenführer, J., and Lengauer, T. (2006). Improved scoring of functional groups from gene expression data by decorrelating GO graph structure. Bioinformatics *22*, 1600–1607.

Benayoun, B.A., Pollina, E.A., Ucar, D., Mahmoudi, S., Karra, K., Wong, E.D., Devarajan, K., Daugherty, A.C., Kundaje, A.B., Mancini, E., et al. (2014). H3K4me3 breadth is linked to cell identity and transcriptional consistency. Cell *158*, 673–688.

Blau, H.M., Chiu, C.P., and Webster, C. (1983). Cytoplasmic activation of human nuclear genes in stable heterocaryons. Cell *32*, 1171–1180.

Blelloch, R., Wang, Z., Meissner, A., Pollard, S., Smith, A., and Jaenisch, R. (2006). Reprogramming efficiency following somatic cell nuclear transfer is influenced by the differentiation and methylation state of the donor nucleus. Stem Cells *24*, 2007–2013.

Boiani, M., Eckardt, S., Schöler, H.R., and McLaughlin, K.J. (2002). Oct4 distribution and level in mouse clones: consequences for pluripotency. Genes Dev. *16*, 1209–1219.

Chung, Y.G., Matoba, S., Liu, Y., Eum, J.H., Lu, F., Jiang, W., Lee, J.E., Sepilian, V., Cha, K.Y., Lee, D.R., and Zhang, Y. (2015). Histone demethylase expression enhances human somatic cell nuclear transfer efficiency and promotes derivation of pluripotent stem cells. Cell Stem Cell *17*, 758–766.

Clouaire, T., Webb, S., Skene, P., Illingworth, R., Kerr, A., Andrews, R., Lee, J.H., Skalnik, D., and Bird, A. (2012). Cfp1 integrates both CpG content and gene activity for accurate H3K4me3 deposition in embryonic stem cells. Genes Dev. *26*, 1714–1728.

Consortium, E.P.; ENCODE Project Consortium (2012). An integrated encyclopedia of DNA elements in the human genome. Nature *489*, 57–74.

Dahl, J.A., Jung, I., Aanes, H., Greggains, G.D., Manaf, A., Lerdrup, M., Li, G., Kuan, S., Li, B., Lee, A.Y., et al. (2016). Broad histone H3K4me3 domains in mouse oocytes modulate maternal-to-zygotic transition. Nature *537*, 548–552.

Enright, B.P., Kubota, C., Yang, X., and Tian, X.C. (2003). Epigenetic characteristics and development of embryos cloned from donor cells treated by trichostatin A or 5-aza-2′-deoxycytidine. Biol. Reprod. *69*, 896–901.

Firas, J., Liu, X., and Polo, J.M. (2014). Epigenetic memory in somatic cell nuclear transfer and induced pluripotency: evidence and implications. Differentiation *88*, 29–32.

Gao, S., Chung, Y.G., Williams, J.W., Riley, J., Moley, K., and Latham, K.E. (2003). Somatic cell-like features of cloned mouse embryos prepared with cultured myoblast nuclei. Biol. Reprod. *69*, 48–56.

Gentsch, G.E., and Smith, J.C. (2014). Investigating physical chromatin associations across the Xenopus genome by chromatin immunoprecipitation. Cold Spring Harb. Protoc. *2014*, 2014.

Gurdon, J.B. (1960). The developmental capacity of nuclei taken from differentiating endoderm cells of Xenopus laevis. J. Embryol. Exp. Morphol. *8*, 505–526.

Gurdon, J.B., Elsdale, T.R., and Fischberg, M. (1958). Sexually mature individuals of Xenopus laevis from the transplantation of single somatic nuclei. Nature *182*, 64–65.

Hirasawa, R., Matoba, S., Inoue, K., and Ogura, A. (2013). Somatic donor cell type correlates with embryonic, but not extra-embryonic, gene expression in postimplantation cloned embryos. PLoS ONE *8*, e76422.

Hörmanseder, E., Tischer, T., and Mayer, T.U. (2013). Modulation of cell cycle control during oocyte-to-embryo transitions. EMBO J. *32*, 2191–2203.

Kim, K., Zhao, R., Doi, A., Ng, K., Unternaehrer, J., Cahan, P., Huo, H., Loh, Y.H., Aryee, M.J., Lensch, M.W., et al. (2011). Donor cell type can influence the epigenome and differentiation potential of human induced pluripotent stem cells. Nat. Biotechnol. *29*, 1117–1119.

Kishigami, S., Mizutani, E., Ohta, H., Hikichi, T., Thuan, N.V., Wakayama, S., Bui, H.T., and Wakayama, T. (2006). Significant improvement of mouse cloning technique by treatment with trichostatin A after somatic nuclear transfer. Biochem. Biophys. Res. Commun. *340*, 183–189.

Lewis, P.W., Müller, M.M., Koletsky, M.S., Cordero, F., Lin, S., Banaszynski, L.A., Garcia, B.A., Muir, T.W., Becher, O.J., and Allis, C.D. (2013). Inhibition of PRC2 activity by a gain-of-function H3 mutation found in pediatric glioblastoma. Science *340*, 857–861.

Li, H., and Durbin, R. (2009). Fast and accurate short read alignment with Burrows-Wheeler transform. Bioinformatics *25*, 1754–1760.

Li, H., Handsaker, B., Wysoker, A., Fennell, T., Ruan, J., Homer, N., Marth, G., Abecasis, G., and Durbin, R.; 1000 Genome Project Data Processing Subgroup (2009). The sequence alignment/map format and SAMtools. Bioinformatics *25*, 2078–2079.

Liu, W., Liu, X., Wang, C., Gao, Y., Gao, R., Kou, X., Zhao, Y., Li, J., Wu, Y., Xiu, W., et al. (2016). Identification of key factors conquering developmental arrest of somatic cell cloned embryos by combining embryo biopsy and single-cell sequencing. Cell Discov. *2*, 16010.

Martin, M. (2011). Cutadapt removes adapter sequences from high-throughput sequencing reads. EMBnet. *17*, 10–12.

Matoba, S., Liu, Y., Lu, F., Iwabuchi, K.A., Shen, L., Inoue, A., and Zhang, Y. (2014). Embryonic development following somatic cell nuclear transfer impeded by persisting histone methylation. Cell *159*, 884–895.

Meissner, A., and Jaenisch, R. (2006). Mammalian nuclear transfer. Dev. Dyn. *235*, 2460–2469.

Muramoto, T., Müller, I., Thomas, G., Melvin, A., and Chubb, J.R. (2010). Methylation of H3K4 is required for inheritance of active transcriptional states. Curr. Biol. *20*, 397–406.

Ng, R.K., and Gurdon, J.B. (2005). Epigenetic memory of active gene transcription is inherited through somatic cell nuclear transfer. Proc. Natl. Acad. Sci. USA *102*, 1957–1962.

Nieuwkoop, P.D., and Faber, J. (1994). Normal Table of Xenopus laevis (Daudin): A Systematical and Chronological Survey of the Development from the Fertilized Egg till the End of Metamorphosis (Garland Pub.).

Polo, J.M., Liu, S., Figueroa, M.E., Kulalert, W., Eminli, S., Tan, K.Y., Apostolou, E., Stadtfeld, M., Li, Y., Shioda, T., et al. (2010). Cell type of origin influences the molecular and functional properties of mouse induced pluripotent stem cells. Nat. Biotechnol. *28*, 848–855.

Probst, A.V., Dunleavy, E., and Almouzni, G. (2009). Epigenetic inheritance during the cell cycle. Nat. Rev. Mol. Cell Biol. *10*, 192–206.

Quinlan, A.R., and Hall, I.M. (2010). BEDTools: a flexible suite of utilities for comparing genomic features. Bioinformatics *26*, 841–842.

Robinson, M.D., McCarthy, D.J., and Smyth, G.K. (2010). edgeR: a Bioconductor package for differential expression analysis of digital gene expression data. Bioinformatics *26*, 139–140.

Robinson, J.T., Thorvaldsdóttir, H., Winckler, W., Guttman, M., Lander, E.S., Getz, G., and Mesirov, J.P. (2011). Integrative genomics viewer. Nat. Biotechnol. *29*, 24–26.

Santos-Rosa, H., Schneider, R., Bannister, A.J., Sherriff, J., Bernstein, B.E., Emre, N.C., Schreiber, S.L., Mellor, J., and Kouzarides, T. (2002). Active genes are tri-methylated at K4 of histone H3. Nature *419*, 407–411.

Takahashi, K., and Yamanaka, S. (2006). Induction of pluripotent stem cells from mouse embryonic and adult fibroblast cultures by defined factors. Cell *126*, 663–676.

Teperek, M., Simeone, A., Gaggioli, V., Miyamoto, K., Allen, G.E., Erkek, S., Kwon, T., Marcotte, E.M., Zegerman, P., Bradshaw, C.R., et al. (2016). Sperm is epigenetically programmed to regulate gene transcription in embryos. Genome Res. *26*, 1034–1046.

Thorvaldsdóttir, H., Robinson, J.T., and Mesirov, J.P. (2013). Integrative Genomics Viewer (IGV): high-performance genomics data visualization and exploration. Brief. Bioinform. *14*, 178–192.

Trapnell, C., Pachter, L., and Salzberg, S.L. (2009). TopHat: discovering splice junctions with RNA-Seq. Bioinformatics *25*, 1105–1111.

STAR★METHODS

KEY RESOURCES TABLE

REAGENT or RESOURCE	SOURCE	IDENTIFIER
Antibodies		
H3K4me3	Abcam	ab8580; RRID: AB_306649
HA	Sigma	H9658; RRID: AB_260092
H4	Abcam	ab31830; RRID: AB_1209246
Chemicals, Peptides, and Recombinant Proteins		
Magnetic beads conjugated with secondary antibody	Invitrogen	11204D
Critical Commercial Assays		
TruSeq RNA library prep kit	Illumina	RS-122-2001
TruSeq DNA kit	Illumina	FC-121-2001
Deposited Data		
Raw and analyzed data	This paper	GEO: GSM733650
Experimental Models: Organisms/Strains		
Xenopus laevis wild type, mature females	Nasco	LM00535MX
Xenopus laevis wild type, mature males	Nasco	LM00715MX
Recombinant DNA		
pCS2+ Kdm5b aa1-770- NLS-6HA	This paper	accession number NM_152895
pCS2+ Kdm5b H499A aa1-770- NLS-6HA	This paper	accession number NM_152895
pCS2+ H3.3-6HA	This paper	accession number NM_001098432
pCS2+ H3.3K4M-6HA	This paper	accession number NM_001098432
Sequence-Based Reagents		
RTqPCR primers (Table S4)	Sigma	NA
Software and Algorithms		
Sickle	https://github.com/najoshi/sickle	https://github.com/najoshi/sickle
cutadapt 1.0	Martin, 2011	https://pypi.python.org/pypi/cutadapt/1.0
TopHat 2.0.6	Trapnell et al., 2009	https://ccb.jhu.edu/software/tophat/index.shtml
BWA (version 0.6.2)	Li and Durbin, 2009	https://sourceforge.net/projects/bio-bwa/files/
samtools 0.1.8	Li et al., 2009	
edgeR	Robinson et al., 2010	https://bioconductor.org/packages/release/bioc/html/edgeR.html
bedtools (version 2.25.0)	Quinlan and Hall, 2010	https://github.com/arq5x/bedtools2/releases
R version 3.2.4	https://www.R-project.org/	https://www.R-project.org/
gplots package version 3.0.1	https://CRAN.R-project.org/package=gplots	https://CRAN.R-project.org/package=gplots

CONTACT FOR REAGENT AND RESOURCE SHARING

Further information and requests for resources and reagents should be directed to and will be fulfilled by the Lead Contact, Eva Hörmanseder e.hoermanseder@gurdon.cam.ac.uk

EXPERIMENTAL MODEL AND SUBJECT DETAILS

Mature *Xenopus laevis* males and females were obtained from Nasco (901 Janesville Avenue, PO Box 901, Fort Atkinson, WI 53538-0901; https://www.enasco.com/xenopus). Our work with *Xenopus laevis* is covered under the Home Office Project License PPL 70/8591 and frog husbandry and all experiments were performed according to the relevant regulatory standards. Animals were maintained in a recirculating fresh water system (Marine Biotech) at a density of one adult/3l, with 10% water change per day and temperatures ranging from 16°C to 20°C. Water was sequentially filtered with mechanical pad sump filter, nitrifying bacteria filter, mechanical canister filter, carbon filter, and UV sterilized. Water quality parameters were as follow: conductivity 1500us; temperature 17-22°C; PH 6-8. Photoperiod was set to 12h ON/12h OFF. Frogs are fed twice per week with Royal Horizon 4.5mm pellets (skretting,

https://www.skrettingfishfeeds.co.uk/). Unconsumed food was removed 10 min after the start of feeding. All material used for this work involves killing of testis-donating frogs by an overdose of anesthetic. Females are injected with hormones (50 units pregnant mare serum gonadotropin, 3 days in advance of egg laying, and 500 units human chorionic gonadotropin, 1 day in advance of egg laying) in the dorsal lymph sack to induce natural ovulation and egg laying in 1xMMR (100mM NaCl, 2mM KCl, 1mM MgSO4, 2mM CaCl2, 0.1mM EDTA, 5mM HEPES (pH 7.8). After egg laying, frogs underwent a health check by a veterinarian and were given a resting period of at least 3 months before re-use. These procedures were of minimal invasiveness and did not cause stress or suffering to the animal. The researchers and the staff of the Gurdon Institute animal husbandry facility are trained in these experiments, and veterinarians monitor the health status of the animals.

METHOD DETAILS

mRNA production

Mouse Kdm5b (accession number NM_152895, aa1-770) and its catalytic inactive (ci) mutant (H499A; aa1-770), both with an C-terminal NLS-tag, as well as *Xenopus* H3.3 (accession number NM_001098432) and its dominant-negative mutant (K4M) constructs were sub-cloned into pCS2+ plasmid with 6 C-terminal HA-tags using the gateway cloning system (Thermo Fisher Scientific). mRNA was synthetized in vitro using MEGAscript SP6 Kit (Ambion, AM1330M) following the manufacturer's instructions.

mRNA injection into one-cell embryos

Eggs were in vitro fertilized, dejellied using 2% Cystein solution in 0.1xMMR, pH 7.8, washed 3 times with 0.1x MMR and transferred into 0.5x MMR for injections. For Kdm5b wild-type and catalytic inactive mutant, 13.6 ng of mRNA was used per injection. For H3.3^{WT} and for H3.3^{K4M} 0.2ng and 1.25ng mRNA, respectively, was used per injection to obtain equal expression levels of the proteins in embryos. Embryos were cultured at 23°C and collected at neurula stage 18 (Kdm5b experiments) or stage 21 (H3.3 experiments) (Nieuwkoop and Faber, 1994) to prepare endoderm donor cells for nuclear transfer.

Donor cell preparation

Endoderm cells were isolated from endoderm tissues of the respective neurula stage embryos (stage 18 for Kdm5b experiments; stage 21 for experiments shown in Figures 1 and 2 or the H3.3 experiments, please also see above) and frozen on dry ice for further analysis or dissociated in calcium- and magnesium-free modified Barth saline (1xMBS; 88mM NaCl, 1mM KCl, 10mM HEPES, 2.5mM NaHCO3, pH to 7.4.) with 1 mM EDTA and 0.1% BSA in a petri dish covered with 1% agarose in H2O and used immediately for nuclear transplantation.

Nuclear transfer and embryo culture

The procedure was carried out as described previously (Gurdon et al., 1958). In brief, dissociated endoderm cells were mildly disrupted by pipetting them up and down gently in a glass micropipette. Nuclear transplantation was performed by injection of a whole permeabilized cell into an egg enucleated for 30 s with a UV mineralite lamp and dejellied by a 5 s Hanovia lamp treatment. Nuclear transfer was performed within the next minute. The nuclear transplant embryos were placed into 1x MBS 0.1% BSA. At the 4-cell stage, the medium was exchanged to 0.1xMBS. As control, embryos were in vitro fertilized and the embryos were cultured at 16°C in 0.1x MBS until they reached stage 7 or stage 11. For all our analyses, completely cleaved NT embryos were selected at stage 7, stage 10 or 11 that were morphologically indistinguishable from IVF embryos at the same stage. For experiments analyzing gene expression at stage 11, NT and IVF embryos with the same blastopore size and cell size were selected to ensure that they are all at the same developmental stage, and the animal cap cells (ectoderm) were isolated and frozen on dry ice for further analysis. To score the developmental outcome, embryos were cultured in 0.1xMBS at 16°C until neurula stage and then at room temperature until they reached the desired developmental stages, which were determined according to the developmental table of Nieuwkoop and Faber (Nieuwkoop and Faber, 1994) and counted.

RNA extraction

Embryonic tissues were selected and isolated as described above, dissected as indicated and frozen at −80°C. RNA extractions were performed using QIAGEN RNeasy Mini kit (QIAGEN, 74106) according to the manufacturer's protocol including the DNase step. RNA was eluted in 40ul of DEPC H2O.

cDNA sequencing library

RNA quality and quantity was analyzed on a RNA screen tape (Agilent) using RNA sample buffer (Agilent) on a Agilent 2200 tape station. Per sample, 500 ng RNA was used to generate a cDNA sequencing libraries using a Illumina TrueSeq kit (RS-122-2001), according to the manufacturer's protocol using 12 PCR amplification cycles.

cDNA synthesis and RTqPCR analysis

cDNA synthesis was performed from the isolated RNAs using oligo dT(15) primers. RTqPCRs were performed with 5 μL cDNA and gene specific primers at 50 nM (Primer sequences are listed in Table S4) using a SybrGreen detection system (Sigma, S9194) and ABI

7300 machine (Applied Biosystems) using standard ABI cycling conditions (two-step PCR cycle: 94°C for 15 s and 60°C for 60 s). Reactions were performed in a total volume of 25 μl.

Western Blotting

Expression of mRNAs in embryos as well as the reduction of H3K4me3 levels was confirmed by western blot analyses. Briefly, embryonic tissues were homogenized in 50 μl buffer E1 (50mM HEPES-KOH pH 7.5, 140mM NaCl, 1mM EDTA pH 8.0, 10% Glycerol, 0.5% Igepal CA-630, 0.25% Triton X-100, 1mM DTT, complete protease inhibitors (Roche)) and then the chromatin was collected by centrifugation at 1600 g for 5 min. The supernatant was kept, the chromatin pellet was washed 3 times with 0.5ml Buffer E1 and then solubilized in 50 μL Emilie's Buffer (500mM Tris pH 6.8, 500mM NaCl, 1% NP40, 0.1% SDS, 1% β-Mercaptoethanol). Laemmli sample buffer was added to the lysate and chromatin fractions, samples were separated on a 15% polyacrylamide gel and transferred to Hybond-P membranes (Amersham Bioscience). Antibodies against H3K4me3 (Abcam ab8580), HA (Sigma, H9658) or histone H4 (Abcam ab31830) were used for western blotting according to standard protocols and suppliers recommendations.

Chromatin Immunoprecipitation (ChiP)

Chromatin Immunoprecipitation (ChIP) were performed as described previously (Gentsch and Smith, 2014) with the following modifications. Neurula (stage 18) embryos were generated by in vitro fertilization. For each ChIP experiment, 50 embryos were dissected in 1xMBS to obtain the endoderm tissue. Samples were fixed in 2 mL of 1% Formaldehyde in 0.1x MMR for 25 min at room temperature, followed by 4 washes with 1 mL 0.1xMMR and equilibration in 500 μL HEG solution (50mM HEPES-KOH pH 7.5, 1 mM EDTA, 20% Glycerol) at 4°C, then excess buffer was removed and samples were frozen at −80°C. To extract chromatin, the samples were homogenized in 2 mL buffer E1 (50mM HEPES-KOH pH 7.5, 140mM NaCl, 1mM EDTA pH 8.0, 10% Glycerol, 0.5% Igepal CA-630, 0.25% Triton X-100, 1mM DTT, complete protease inhibitors (Roche)). Chromatin was collected by centrifugation for 2 min at 3500 rmp, 4°C and then washed two times with 2 mL E1, three times with 2ml buffer E2 (10mM Tris pH 8.0, 200mM NaCl, 1mM EDTA, 0.5mM EGTA, complete protease inhibitors (Roche)) and three times with 500 μL Buffer E3 (10mM Tris pH 8.0, 200mM NaCl, 1mM EDTA, 0.5mM EGTA, 0.1% Na-deoxycholate, 0.5% N-lauroylsarcosine, complete protease inhibitors (Roche)). Chromatin was fragmented by sonication for 20 cycles (30 s on and 30 s off) using a Bioruptor (Diagenode) at 4°C. The samples were centrifuged at 15min, 4°C at full speed, the supernatant was collected and Triton X-100 was added to 1%. 25 μL of the solution were put aside to serve as Input for later analysis. Before ChIP, primary anti H3K4me3 (Abcam ab8580, 0.5 μg per 50 embryos) antibodies were bound to PBS washed magnetic beads conjugated with secondary antibody (Invitrogen 11204D, 25 μL per 50 embryos) in 500 μL 1xPBS 0.1% BSA overnight at 4°C on a rotating wheel. Beads were washed 3 times with 1xPBS 0.1% BSA, added to the fragmented chromatin solution and incubated overnight at 4°C on a rotating wheel. Beads were then washed 6 times with RIPA buffer (50mM HEPES-KOH pH 7.5, 500mM LiCl, 1mM EDTA, 1% Igepal CA-630, 0.7% Na-deoxycholate, complete protease inhibitors (Roche)) and twice with TEN buffer (10mM Tris pH 8.0, 1mM EDTA, 150mM NaCl, complete protease inhibitors (Roche)) for each 10 min. For crosslink reversal, the beads were resuspended in 150 μL Stop buffer (40mM Tris pH 8.0, 10mM EDTA, 1% SDS) and 125 μL Stop buffer was added to the input fraction. The samples were supplemented with Proteinase K (0.3 μg/μl), NaCl (250 mM) and incubated at 65°C overnight. RNase A (DNase free) was added to a final concentration of 200 μg/μl and DNA was Phenol/Chloroform extracted. 150 μg/μl Glycogen was added and DNA was recovered by Ethanol precipitation. The pellet was resuspended in 30 μL H2O.

ChIP-seq library preparation

Half of a ChIP reaction (15 μl, see above) were subjected for ChIP-seq library preparation with the TruSeq DNA kit (Illumina, FC-121-2001). Two independent biological replicates were generated for each H3K4me3 ChIP experiments.

ChIP-RTqPCR

The ChIP reaction (see above) was diluted 1:40 and 5 μL were used for subsequent RTqPCR analysis using primer pairs described in Table S4 at 50nM together with a SybrGreen detection system (Sigma, S9194) and ABI 7300 machine (Applied Biosystems) using standard ABI cycling conditions (two-step PCR cycle: 94°C for 15 s and 60°C for 60 s). Reactions are performed in a total volume of 25 μl.

Experimental design

In all experiments analyzing gene expression, one sample was taken per embryo (i.e., one sample corresponds to one individual embryo). For the analysis of ON-memory gene-expression, 3 independent experiments (here defined as 3 biological replicates) were performed. In experiment#1, 4 IVF-, 3 NT-ectoderm samples and 1 endoderm donor sample, in experiment#2, 4 IVF-, 4 NT-ectoderm samples and 1 endoderm donor sample, in experiment#3, 3 IVF-, 5 NT-ectoderm samples and 1 endoderm donor sample were generated (see Table S1). To address the effect of Kdm5b, 2 independent experiments (here defined as two biological replicates) were performed. In experiment#4, 4 NT(Kdm5b^{wt})- and 4 NT(Kdm5b^{ci}) - ectoderm samples and 2 NT(Kdm5b^{wt})- and 2 NT(Kdm5b^{ci})- endoderm donor cell samples and in experiment#5, 3 NT(Kdm5b^{wt})- and 4 NT(Kdm5b^{ci}) - ectoderm samples and 2 NT(Kdm5b^{wt})- and 2 NT(Kdm5b^{ci})- endoderm donor cell samples were taken (see Table S1). For the H3.3^{K4M} analysis, one experiment#6 (one biological replicate) was performed with 4 IVF ectoderm samples, 4 NT(H3.3^{wt})- and 4 NT(H3.3^{K4M})- ectoderm samples and 2 NT(H3.3^{wt})- and 2 NT(H3.3^{K4M}) - endoderm donor cell samples.

Randomization and Blinding: When the donor cells were treated, the order of nuclear transfer of control- and treated donor nuclei was alternated; The experimenter was unaware of the treatment of the donor cell while performing the nuclear transfer. When embryos were selected for analysis, healthy looking embryos (i.e., morphologically indistinguishable from IVF-embryos) with the same blastopore size were selected. A sample size was chosen that allowed the significant identification of differentially expressed genes (see quantification and statistical data analysis section below), and that also considered the loss of a third of the samples due to inefficient RNA extraction or a failure in library generation. Samples were excluded that showed poor RNA quality (RIN below 7), quantity (below 500ng) or that did not result in a product after performing the library preparation protocol. Furthermore, after sequencing, the raw reads were clustered using WardD, and out of 6 experiments, 3 experiments contained outliers: exp#1: two NT-samples; exp#3: one IVF-sample and exp#5: one NT(Kdm5b^{wt})- sample. These 4 samples were excluded from further DE gene expression analysis.

For ChIP experiments, 2 independent experiments (here referred to as 2 biological replicates) were performed. Per ChIP experiment, the endoderm tissues of 50 Kdm5b^{wt}-, 50 Kdm5b^{ci}- expressing embryos, as well as 50 uninjected embryos were pooled.

For the quantification of the developmental outcome, 6 independent experiments (n) were performed, and the total number of gastrulae (N) was determined for each condition: 69 NT(Kdm5b^{ci})- and 56 NT(Kdm5b^{ci})- and 152 IVF- gastrula embryos. Gastrula embryos were selected, that were morphologically indistinguishable from IVF embryos at the same stage. Here, the biological replicates refer to the number of gastrula embryos quantified. Their development was followed, and surviving embryos were counted for each condition at the indicated stages. Randomization and Blinding: Per experiment, each condition was performed and the order was alternated; The experimenter was unaware of the treatment of the donor cell while performing the nuclear transfer and the quantification of the survival.

QUANTIFICATION AND STATISTICAL ANALYSIS

Xenopus transcriptome and sequencing data

We used the *Xenopus laevis* annotation that was generated for (Teperek et al., 2016). RNA-seq and ChIP-seq libraries were sequenced on an Illumina HiSeq 2000 instrument in single read mode at 36 base length. Fastq files were filtered for low quality reads (< Q20) using sickle and low quality bases were trimmed from the ends of the reads (< Q20). Adapters were removed using cutadapt 1.0 (Martin, 2011). RNA-seq data were mapped against the *Xenopus laevis* genome using TopHat 2.0.6 (Trapnell et al., 2009) - *Xenopus laevis* genome (JGI version 6.1) was used for all analyses in this paper, and can be downloaded here: ftp://ftp.xenbase.org/pub/Genomics/JGI/Xenla6.1/.). ChIP-seq data mapped against *X. laevis* (version 6.1) with BWA (version 0.6.2) (Li and Durbin, 2009). Duplicate reads in ChIP-seq were then filtered out with samtools 0.1.8 (Li et al., 2009). After this step, our input dataset contained more than 18 M uniquely mapped reads, and our IP samples more than 10 M uniquely mapped reads.

Xenopus differential gene expression

For the expression profiling, read counts were generated for each of the transcripts. RPKMs (reads per kilobase per million) were calculated by normalizing read counts for each transcript by the transcript length and the total number of reads in the corresponding sample. Counts per million (CPM) and differentially expressed (DE) transcripts were called using edgeR (Robinson et al., 2010). For the analysis of the extent of transcriptional memory in Figures 1 and 2, transcripts remained in the analysis if they had CPM > 1 in either all of the Donor or 70% of IVF or 70% of NT embryo samples. The log2 fold change (logFC) and the false discovery rate (FDR) was calculated comparing 11 IVF ectoderm samples, 12 NT ectoderm samples and 3 endoderm donor cell samples from 3 independent experiments (see Table S1). To address the effect of Kdm5b, the DE analysis was performed on 8 IVF ectoderm samples, 7 NT(Kdm5b^{wt})- and 8 NT(Kdm5b^{ci}) - ectoderm samples and 4 NT(Kdm5b^{wt})- and 4 NT(Kdm5b^{ci})- endoderm donor cell samples from 2 independent experiments (see Table S1). The H3.3^{K4M} DE analysis was performed on 4 IVF ectoderm samples, 4 NT(H3.3^{wt})- and 4 NT(H3.3^{K4M})- ectoderm samples and 2 NT(H3.3^{wt})- and 2 NT(H3.3^{K4M}) - endoderm donor cell samples from one experiment.

Human differential gene expression

Publicly available datasets of the gene expression analysis were obtained from the Gene Expression Omnibus (GEO) DataSets, accession number GSE73362 (human) (Chung et al., 2015). The logFCs in gene expression levels were calculated in R.

DE data filter-strategy

X.Laevis, log2 fold changes (logFC) and false discovery rate (FDR) were calculated by using the R package EdgeR. These lists of transcripts were then additionally filtered the following way (note that in the *Xenopus* analysis "3FC" corresponds log2FC < 1.5 or log2FC > 1.5, which is more precisely a 2.8285 fold change, and approximately a 3 fold change):

DE transcripts Donor/IVF: $FDR^{Donor/IVF} < 0.05$; DE between Donor/IVF and NT/IVF: $FDR^{Donor/IVF} < 0.05$&$FDR^{NT/IVF} < 0.05$. ON-memory: $FDR^{Donor/IVF} < 0.05$, $logFC^{Donor/IVF} > 0$, $FDR^{NT/IVF} < 0.05$, $logFC^{NT/IVF} > 0$, $RPKM^{Donor} > 1$; ON-memory(3FC): $FDR^{Donor/IVF} < 0.05$, $logFC^{Donor/IVF} > 0$, $FDR^{NT/IVF} < 0.05$, $logFC^{NT/IVF} > 1.5$, $RPKM^{Donor} > 1$; OFF-memory: $FDR^{Donor/IVF} < 0.05$, $logFC^{Donor/IVF} < 0$, $FDR^{NT/IVF} < 0.05$, $logFC^{NT/IVF} < 0$; OFF-memory(3FC): $FDR^{Donor/IVF} < 0.05$, $logFC^{Donor/IVF} < 0$, $FDR^{Donor/NT} < 0.05$, $logFC^{Donor/NT} < 0$, $FDR^{NT/IVF} < 0.05$, $logFC^{NT/IVF} < -1.5$; Reprogrammed-down: $FDR^{Donor/IVF} < 0.05$, $logFC^{Donor/IVF} > 0$, $FDR^{Donor/NT} < 0.05$, $logFC^{Donor/NT} > 0$, $RPKM^{Donor} > 1$; transcripts with $FDR^{NT/IVF} < 0.05$ were excluded. Note that transcripts that were transcribed in the Donor (RPKM > 1 in all Donor samples) but not in IVF and NT (RPKM < 1 in some or all samples) were kept in the analysis and considered as ON-reprogrammed as they were

successfully downregulated during reprogramming. Reprogrammed-up: $FDR^{Donor/IVF} < 0.05$, $logFC^{Donor/IVF} < 0$, $FDR^{Donor/NT} < 0.05$, $logFC^{Donor/NT} < 0$, $FDR^{NT/IVF} > 0.05$. Reprogrammed: $FDR^{Donor/IVF} < 0.05$, $FDR^{Donor/NT} < 0.05$, transcripts with $FDR^{NT/IVF} < 0.05$ were excluded.

Gene ontology terms over-represented among the differentially expressed genes were found using topGO (Alexa et al., 2006).

In the Kdm5b experiments, each of the two experiments was filtered separately and then the generated lists were intersected (Figures 3, S3, and S4; Tables S2 and S3). DE transcripts between $Kdm5b^{wt}$ versus $Kdm5b^{ci}$ expressing donor and $H3.3^{wt}$ versus $H3.3^{K4M}$ expressing donor cells were identified by filtering for FDR < 0.05 and then excluded from further analysis. The different gene sets upon $Kdm5b^{ci}$ treatment were filtered the following way:

DE transcripts $Donor^{ci}$/IVF: $FDR^{Donorci/IVF} < 0.05$; DE between $Donor^{ci}$/IVF and NT^{ci}/IVF: $FDR^{Donorci/IVF} < 0.05$&$FDR^{NTci/IVF} < 0.05$. ON-memory: $FDR^{Donorci/IVF} < 0.05$, $logFC^{Donorci/IVF} > 0$, $FDR^{NTci/IVF} < 0.05$, $logFC^{NTci/IVF} > 0$, $RPKM^{Donorci} > 1$; ON-memory(3FC): $FDR^{Donorci/IVF} < 0.05$, $logFC^{Donorci/IVF} > 0$, $FDR^{NTci/IVF} < 0.05$, $logFC^{NTci/IVF} > 1.5$, $RPKM^{Donocir} > 1$; OFF-memory: $FDR^{Donorci/IVF} < 0.05$, $logFC^{Donorci/IVF} < 0$, $FDR^{NTci/IVF} < 0.05$, $logFC^{NTci/IVF} < 0$; OFF-memory(3FC): $FDR^{Donorci/IVF} < 0.05$, $logFC^{Donorci/IVF} < 0$, $FDR^{Donorci/NT} < 0.05$, $logFC^{Donorci/NT} < 0$, $FDR^{NTci/IVF} < 0.05$, $logFC^{NTci/IVF} < -1.5$; Reprogrammed-down: $FDR^{Donorci/IVF} < 0.05$, $logFC^{Donorci/IVF} > 0$, $RPKM^{Donorci} > 1$; transcripts with $FDR^{NT/IVF} < 0.05$ were excluded. Note that transcripts that were transcribed in the Donor (RPKM > 1 in all Donor samples) but not in IVF and NT (RPKM < 1 is some or all samples) were kept in the analysis and considered as ON-reprogrammed as they were successfully downregulated during reprogramming. Reprogrammed-up: $FDR^{Donorci/IVF} < 0.05$, $logFC^{Donorci/IVF} < 0$, $FDR^{NTci/IVF} > 0.05$. Reprogrammed: $FDR^{Donorci/IVF} < 0.05$, transcripts with $FDR^{NTci/IVF} < 0.05$ were excluded

The different gene sets upon $Kdm5b^{wt}$, $H3.3^{wt}$ and $H3.3^{K4M}$ treatment were filtered following the same strategy as above.

In human and mouse, the values for log2 FC (logFC) were filtered using R. These lists of transcripts were then additionally filtered the following way:

DE transcripts between Donor/IVF: $FC^{Donor/IVF} > 5$; DE between Donor/IVF and NT/IVF: $FC^{Donor/IVF} > 5$ and $FC^{NT/IVF} > 5$. ON-memory(2-5FC): $logFC^{Donor/IVF} > 2.3$, $1 < logFC^{NT/IVF} < 2.3$, $RPKM^{Donor} > 1$; ON-memory(5FC):, $logFC^{Donor/IVF} > 2.3$, $logFC^{NT/IVF} > 2.3$, $FPKM^{Donor} > 1$; OFF-memory(2-5FC): $logFC^{Donor/IVF} < -2.3$, $-2.3 < logFC^{NT/IVF} < -1$; OFF-memory(5FC): $logFC^{Donor/IVF} < -2.3$, $logFC^{NT/IVF} < -2.3$; Reprogrammed-down: $logFC^{Donor/IVF} > 2.3$, $RPKM^{Donor} > 1$; transcripts with $logFC^{NT/IVF} > 1$ were excluded. Note that genes that were transcribed in the Donor (FPKM > 1 in all Donor samples) but not in IVF and NT (FPKM < 1 in some or all samples) were kept in the analysis and considered as ON-reprogrammed as they were successfully downregulated during reprogramming. Reprogrammed-up: $logFC^{Donor/IVF} < -2.3$, $logFC^{NT/IVF} > -1$. Reprogrammed: union of ON- and OFF- reprogrammed transcripts.

Heatmaps and plots for gene expression

Heatmaps. The log2 fold change was calculated over the mean IVF expression level or over the mean pooled donor, IVF and NT expression level, as indicated in figure legends. These values were plotted on a heatmap and clustered by rows only or by both rows and columns, as shown in figure legends, using heatmap.2 (from R package *gplots*) using default settings (which is complete as agglomeration method and Euclidean distance as similarity measure). MA plot. The log2 FC in expression of transcripts between NT- to IVF-embryos was plotted against the average donor cell gene expression (log2(RPKM+1)). Box-plots show distribution of mean gene expression levels of the different sets of transcripts. The middle line in the box indicates the median, the box edges indicate the 25th/75th percentiles, the whiskers indicate the min and max.

Differences in gene expression levels between pairwise sets of genes were tested using Mann-Whitney test (equivalent to Wilcoxon rank sum test. R, wilcox.test(alternative = c("two.sided"), paired = F)).

qPCR analysis

The indicated genes were quantified using a standard curve of embryonic cDNA (gene expression) or *Xenopus* genomic DNA (ChIP). For normalization, the values of the genes of interest were divided by the values for H4 (gene expression) or the values of the IP were represented as percent of the Input values (ChIP). The data were then visualized using R as a scatterplot, with the mean and the standard error of the mean (gene expression); each dot on the scatterplot corresponds to one embryo sample generated in two independent experiments) or the mean as a column bar graph with the standard error of the mean (ChIP; each bar corresponds to two values generated in two independent experiments). When indicated, significance was calculated using Mann-Whitney test (equivalent to Wilcoxon rank sum test. R, wilcox.test(alternative = c("two.sided"), paired = F)). *p value < 0.05, **p value < 0.01, ***p value < 0.001.

Principal component analysis and hierarchical transcriptome clustering

After computing CPM (count per million), genes were retained in the analysis if they had CPM > 1 in either all of the Donor or in $\geq$ 70% of IVF or $\geq$ 70% of NT embryo samples. The data were subsequently scaled two times using z-score transformation: one scaling has been performed for each batch of experiments (i.e., experiments produced at the same time). Then all batched experiments have been scaled together again to reduce the variability due to the technical batch factor. Data obtained after this step have been used for the unsupervised hierarchical clustering analysis, which was performed by using the Euclidean distance and ward.D linkage as implemented in R. The PCA analysis was performed using the R function prcomp() using the parameter cor = T.x.

ChIP-seq data analysis

Aligned data from H3K4me3 ChIP-seq were used to compute the coverage around TSS (transcriptional start site) for each of the two biological replicates separately. The histone methylation levels was computed as:

$$Histone\ Methylation\ level = \frac{Coverage_{IP}}{N_{IP}}10^6 - \frac{Coverage_{input}}{N_{input}}10^6$$

where IP is the immunoprecipitation sample, *input* is the input-control; N_{IP} is the total number of aligned reads in the IP experiment and N_{input} is the total number of aligned reads in the input sample.

Specifically, a region of 4kb centered on the TSSs was binned in 50bp-wide windows and the histone methylation level was computed for each bin. The average of the normalized histone methylation levels was computed for each set of genes (ON-memory, ON-reprogrammed, genome-wide (GW)) and then visualized. Differences in histone methylation levels at TSSs between the set of genes were tested with the Kolmogorov-Smirnov test (R, ks.test).

Additionally, the global (integral) histone methylation level in the 4kb window was computed and the distribution across each set of genes was visualized in box-plots and compared using ks.test. For signal tracks, count reads were computed with bedtools (version 2.25.0) genomecov (Quinlan and Hall, 2010).

Processed and normalized bigwig files relative to H3K4me3 in adult normal human dermal fibroblast (NHDF) cells were downloaded from GEO (GEO accession number GSM733650). The same strategy was used to compare the average histone methylation levels of different groups of genes in a region of 2kb around TSS as indicated.

Methylated histone regions

Histone methylated regions (peaks) were called using MACS2 (version 2.0.9) with the following options (–broad–gsize = 2.6e9 -q 0.01) for each immunoprecipitation experiment individually. Resulting peaks overlapping TSSs were used for the subsequent analysis. Peaks sizes distributions were visualized by plotting histograms. The inserts with examples of H3K4me3 regions spanning the TSS of example genes were generated using Integrative Genomics Viewer, IGV (Robinson et al., 2011; Thorvaldsdóttir et al., 2013). KS test was used to evaluate differences between peaks sizes across the different set of previously defined genes. ECDFs (empirical cumulative distribution functions) were computed for all experiments and visualized.

We downloaded peaks list of the human study in NHDF cells (GEO accession number GSM733650). Peak sizes distributions of different set of genes were compared similarly.

Developmental outcome

Statistical significance was calculated using a one tailed t test using R. The data are represented as the mean with the SEM (standard error of the mean).

DATA AVAILABILITY

The RNA-seq and ChIP-seq data generated in this study are: 73 samples, single-ended RNA-seq libraries from neurula stage 18 or 21 endoderm and gastrula stage 11 ectoderm samples; 2 single-ended ChIP-seq libraries from endoderm cells of neurula (stage 21) embryos with antibody for H3K4me3, and 2 replicates for each histone modification pull-down. The accession number for the RNA-seq and ChIP-seq data reported in this paper is GEO: GSE92366.

Cell Stem Cell, Volume *21*

Supplemental Information

H3K4 Methylation-Dependent Memory of Somatic Cell Identity Inhibits Reprogramming and Development of Nuclear Transfer Embryos

Eva Hörmanseder, Angela Simeone, George E. Allen, Charles R. Bradshaw, Magdalena Figlmüller, John Gurdon, and Jerome Jullien

Figure S1

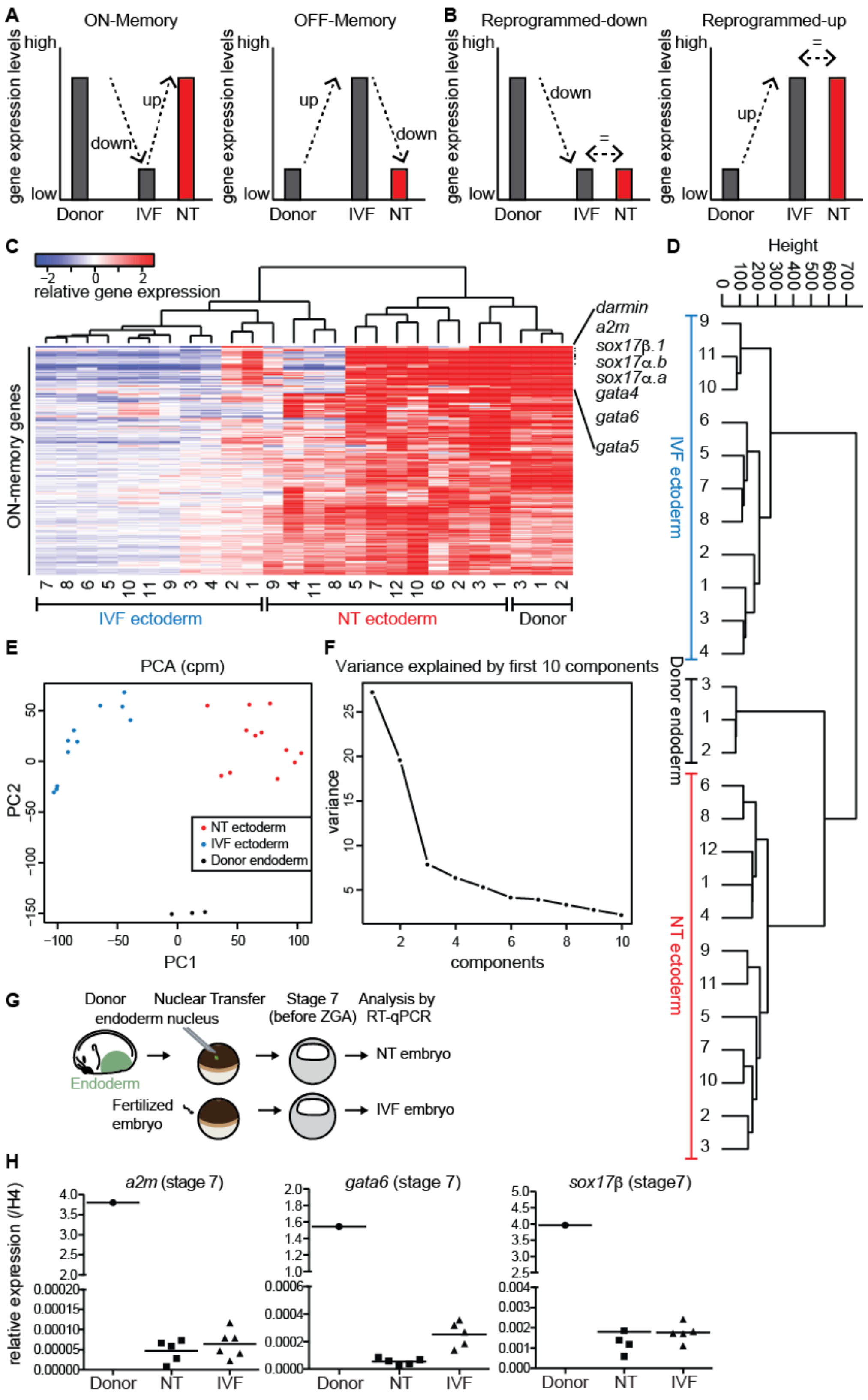

Figure S1. Related to Figure 1; Changes from donor endoderm cells to NT embryos ectoderm cells: Memory and reprogramming of gene expression. (A) The gene expression in the donor endoderm cells was compared with gene expression in the ectoderm cells of control IVF embryos. This revealed genes that are differentially expressed between the two cell-types. Next, gene expression between the ectoderm cells of control IVF embryos and the ectoderm cells of NT embryos was compared. This revealed genes that are differentially expressed between IVF and NT

ectoderm cells and thus represent reprogramming resistant genes. The group of reprogramming resistant genes comprises ON-memory genes, which are genes that were expressed in the endoderm donor cells and are down-regulated in ectoderm cells of IVF embryos, but remain up-regulated in the ectoderm cells of NT embryos. Furthermore, the group of reprogramming resistant genes also contains OFF-memory genes. These are genes that are up-regulated in IVF ectoderm when compared to endoderm donor cells, but remain down-regulated in NT ectoderm cells. **(B)** Instead genes that are differentially expressed between the endoderm donor cells and the IVF ectoderm cells and that were similarly expressed in the IVF and NT ectoderm cells represent successfully reprogrammed genes. **(C)** All NT embryos show genes with an active state of gene-expression (ON-memory). Heatmap illustration comparing ON-memory(3FC) gene expression in ectoderm tissues of single (not pooled) IVF and NT embryos as well as donor endoderm cells. Rows and columns are sorted by hierarchical clustering (agglomeration method: complete, Euclidian distance function). Examples of endoderm lineage genes showing ON-memory are indicated. **(D-F)** Filtered and normalized RNAseq data of single ectoderm tissues of IVF and NT embryos as well as donor endoderm cells presented in Fig.1 and 2. **(D)** Hierarchical transcriptome clustering analysis (agglomeration method: Ward.D as implemented in R, Euclidean distance function) **(E)** Principal component analysis (PCA). First two principal components (which explain 27% and 16.5% of the variance) were computed using the R function prcomp() with the parameter cor = T. **(F)** Percentage of variance explained by the first 10 principal components of data shown in (E). (G) Endoderm donor specific genes are not detected before zygotic genome activation – design of NT experiments. After NT of an endoderm donor nucleus to an enucleated egg, stage 7 embryos (prior to zygotic genome activation, ZGA) were collected. As controls, eggs were fertilized and collected at the same stage. (H) Donor endoderm-cells as well as NT and IVF ectoderm cells were analysed by RT-qPCR for *a2m*, *gata6* and *sox17β* relative to *H4* in whole stage 7 embryos. NT, nuclear transfer; IVF, *in vitro* fertilized; RT-qPCR, quantitative real-time PCR;

Figure S2

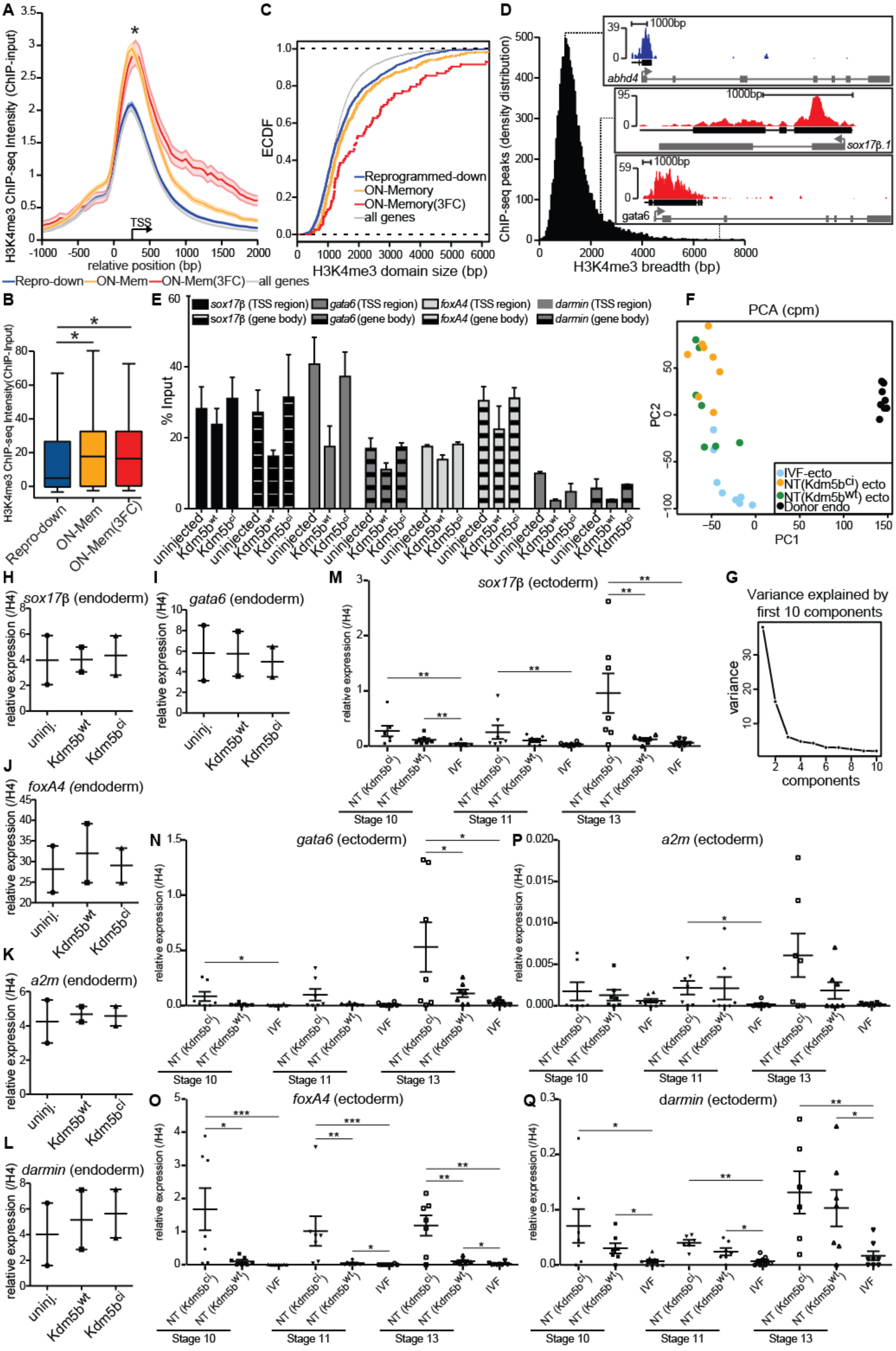
A
H3K4me3 ChIP-seq Intensity (ChIP-input)
TSS
relative position (bp)
Repro-down
ON-Mem
ON-Mem(3FC)
all genes
B
H3K4me3 ChIP-seq Intensity(ChIP-Input)
Repro-down
ON-Mem
ON-Mem(3FC)
C
ECDF
Reprogrammed-down
ON-Memory
ON-Memory(3FC)
all genes
H3K4me3 domain size (bp)
D
ChIP-seq peaks (density distribution)
H3K4me3 breadth (bp)
1000bp
abhd4
sox17β.1
gata6
E
sox17β (TSS region)
gata6 (TSS region)
foxA4 (TSS region)
darmin (TSS region)
sox17β (gene body)
gata6 (gene body)
foxA4 (gene body)
darmin (gene body)
% Input
uninjected
Kdm5bwt
Kdm5bci
F
PCA (cpm)
PC1
PC2
IVF-ecto
NT(Kdm5bci) ecto
NT(Kdm5bwt) ecto
Donor endo
G
Variance explained by first 10 components
variance
components
H
sox17β (endoderm)
I
gata6 (endoderm)
J
foxA4 (endoderm)
K
a2m (endoderm)
L
darmin (endoderm)
relative expression (/H4)
uninj.
Kdm5bwt
Kdm5bci
M
sox17β (ectoderm)
N
gata6 (ectoderm)
O
foxA4 (ectoderm)
P
a2m (ectoderm)
Q
darmin (ectoderm)
NT (Kdm5bci)
NT (Kdm5bwt)
IVF
Stage 10
Stage 11
Stage 13

Figure S2. Related to Figure 2 and 3; ON-memory genes are enriched for H3K4me3 when compared to reprogrammed-down genes in *Xenopus* endoderm donor cells and Kdm5b treatment of donor reduces ON-memory gene expression in the resulting NT embryos.

(A-D) H3K4me3 ChIP-seq data was generated from endoderm cells of neurula-stage embryos as used for NT experiments; second biological replicate is shown here. Read counts are normalized by input and total mapped reads. **(A)** TSS metaplot of the average intensity of H3K4me3 modifications in endoderm cells are shown for reprogrammed-down, ON-memory genes, ON-memory(3FC) and all genes from the *Xenopus* genome. ON-memory(3FC) and ON-memory ChIP-seq intensities are higher when compared to reprogrammed-down genes (p-value= 0.071 and *p-value= 0.0006, respectively; 4 kb window, KS-test). **(B)** ON-memory genes when compared to reprogrammed-down genes, show increased H3K4me3 levels in the donor cells. Box plot comparing mean H3K4me3 ChIP-seq intensities of reprogrammed-down, ON-memory and ON-memory(3FC) in a 4kb window centred on the TSS (*p-value< 0.001, KS-test). **(C)** Empirical cumulative distribution function comparing H3K4me3 domain size around the TSS of reprogrammed-down, ON-memory genes, ON-memory(3FC), and all genes from the *Xenopus* genome. ON-memory(3FC) genes show a significant increase in H3K4me3 breadth when compared to reprogrammed-down genes (p-value= 8.55E-07, KS-test; ChIP-seq peaks called by MACS2). **(D)** Breadth distribution of H3K4me3 ChIP-seq peaks called by MACS2. Inserts are examples of H3K4me3 regions of a reprogrammed-down gene (*abhd4*) and two ON-memory(3FC) genes, *sox17β.1* and *gata6* (NM_001087983.1). **(E)** ChIP-RTqPCR verification of the reduction in H3K4me3 levels upon Kdm5b^{wt} treatment on candidate ON-memory genes. ChIP-RTqPCR showing H3K4me3 enrichment over *sox17β*, *gata6*, *foxa4* and *darmin* TSS and gene body regions in endoderm cells isolated from uninjected, Kdm5b^{wt} and Kdm5b^{ci} expressing stage 18 embryos (n=2). Data are presented as mean ± SEM. TSS, transcriptional start site. **(F)** Principal component analysis (PCA) of filtered and normalized RNAseq data of single ectoderm tissues of IVF and NT embryos as well as donor endoderm cells, see Fig.3. First two principal components (which explain 27% and 16.5% of the variance) were computed using the R function prcomp() with the parameter cor = T. **(G)** Percentage of variance explained by the first 10 principal components of data shown in (F). **(H-Q)** Reduction of H3K4 methylation via Kdm5b^{wt} in donor cells via expression of H3.3^{K4M} reduces expression of some ON-memory genes in the resulting NT embryos throughout gastrulation. In two independent experiments, the expression of candidate memory genes (*sox17β*, *gata6*, *foxa4*, *a2m* and *darmin*) was assessed by RT-qPCR in **(H-L)** the endoderm donor cells and **(M-Q)** in the ectoderm cells of 7 NT(Kdm5b^{ci}) , 7 NT(Kdm5b^{wt}) and 8 IVF embryos (single, not pooled) at different stages during gastrulation. Increased candidate memory gene expression can be observed in all treatment control NT(Kdm5b^{ci}) embryos when compared to the IVF–embryos. Candidate ON-memory gene expression is reduced upon treatment of the donor cell with Kdm5b^{wt} and for some genes this effect is more pronounced (*sox17β*, *gata6*, *foxa4*) than for others (*a2m* and *darmin*). * p ≤ 0.05, ** p ≤ 0.01, *** p ≤ 0.001; data are presented as mean ± SEM. Box plots: middle line in the box indicates the median, the box edges indicate the 25th/75th percentiles, the whiskers indicate the min and max.

Figure S3

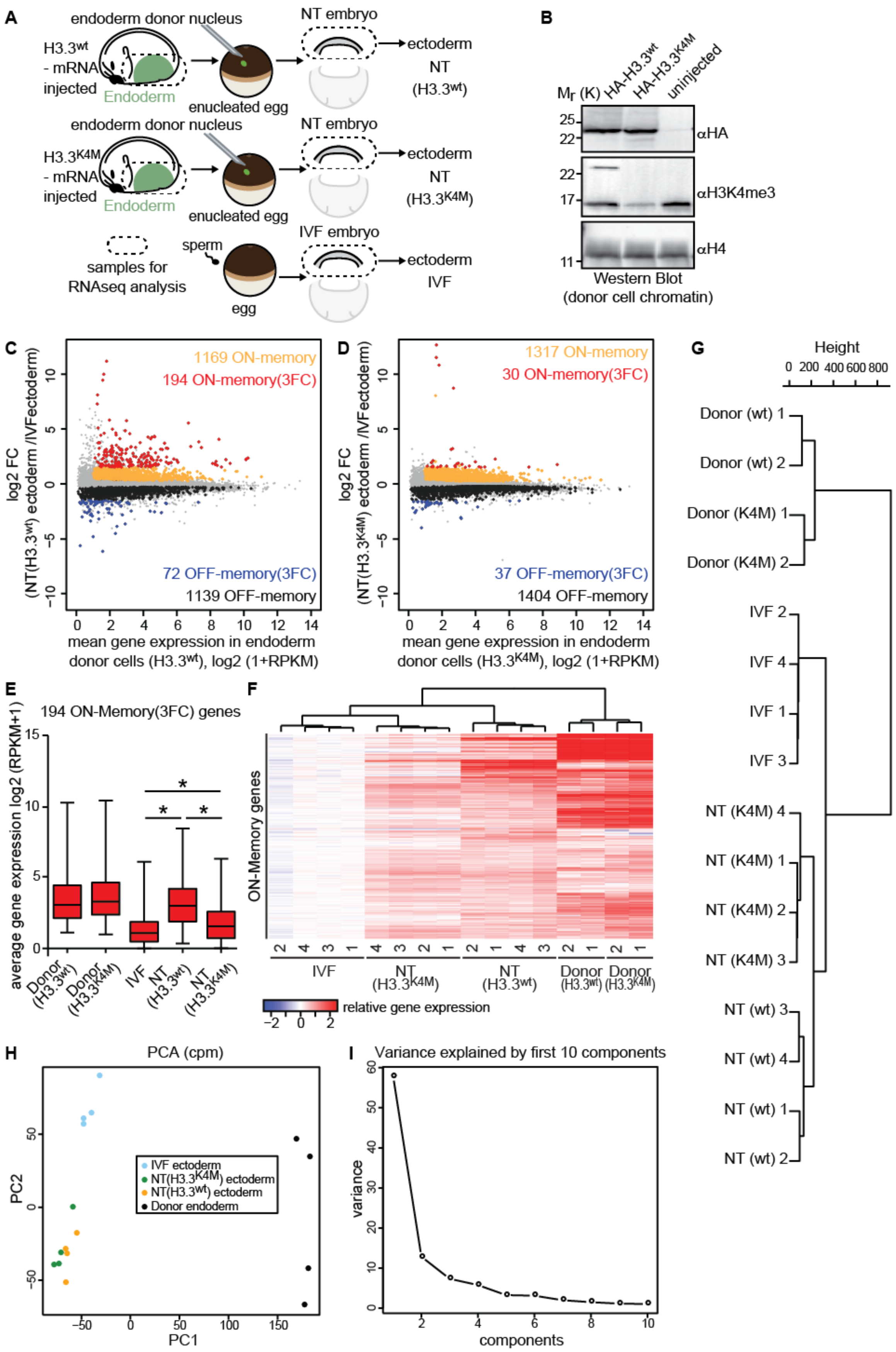

Figure S3. Related to Figure 3; Inhibition of H3K4me3 specific methyltransferases in the donor cells via expression of H3.3^{K4M} reduces ON-memory gene expression in the resulting NT embryos. (A) Design of NT experiments. After NT of an endoderm donor nucleus (expressing H3.3^{K4M} or H3.3^{wt}) to an enucleated egg, gastrula embryos were collected. As controls, IVF

embryos were collected at the same stage. The endoderm was isolated from donor embryos, the ectoderm was isolated from NT and IVF embryos, and all tissues were analysed by RNA-seq. **(B)** Western Blot analysis showing that H3.3^{K4M}, but not to H3.3^{wt} expression reduces H3K4me3 levels to ≈75% of control (uninjected) levels in neurula stage embryos. **(C-F)** H3.3^{K4M} expression in the donor cells reduces the number of miss-regulated genes in NT embryos when compared to IVF embryos. MA plot comparing gene expression between ectoderm cells of **(C)** NT(H3.3^{wt}) and IVF embryos or **(D)** NT(H3.3^{K4M}) and IVF embryos. The average log2 fold change in expression of transcripts in ectoderm cells of NT embryos over IVF embryos was plotted on the y axis, the mean log2 (1+RPKM) gene expression in the endoderm donor cells was plotted on the x axis (ectoderm of 4 NT(H3.3^{K4M}), 4 NT(H3.3^{wt}) and 4 IVF embryos; 2 endoderm tissues of H3.3^{K4M} - or H3.3^{wt} - expressing embryos. n=1, see Tab.S1). Gray, all identified transcripts; orange, ON-memory and black, OFF-memory; red, ON-memory genes; blue, OFF-memory genes. **(E)** Box plots comparing the mean expression levels (RPKM) of ON-memory transcripts in endoderm donor cells and the ectoderm tissues of IVF , NT(H3.3^{wt}) and NT(H3.3^{K4M}) embryos. (*p-values<0.001) **(F)** Heatmap illustration comparing ON-memory gene expression in single ectoderm tissues of IVF, NT(H3.3^{K4M}) and NT(H3.3^{WT}) embryos as well as in the endoderm donor cells. Rows and columns are sorted by hierarchical clustering. For detailed numbers see Table S5. **(G)** Hierarchical transcriptome clustering analysis (agglomeration method: Ward.D as implemented in R, Euclidean distance function) and **(H)** Principal component analysis (PCA) of filtered and normalized RNAseq data of single ectoderm tissues of IVF and NT embryos as well as donor endoderm cells presented in this figure. First two principal components (which explain 27% and 16.5% of the variance) were computed using the R function prcomp() with the parameter cor = T. **(I)** Percentage of variance explained by the first 10 principal components of data shown in (H).
Box plots: middle line in the box indicates the median, the box edges indicate the 25th/75th percentiles, the whiskers indicate the min and max.

Figure S4

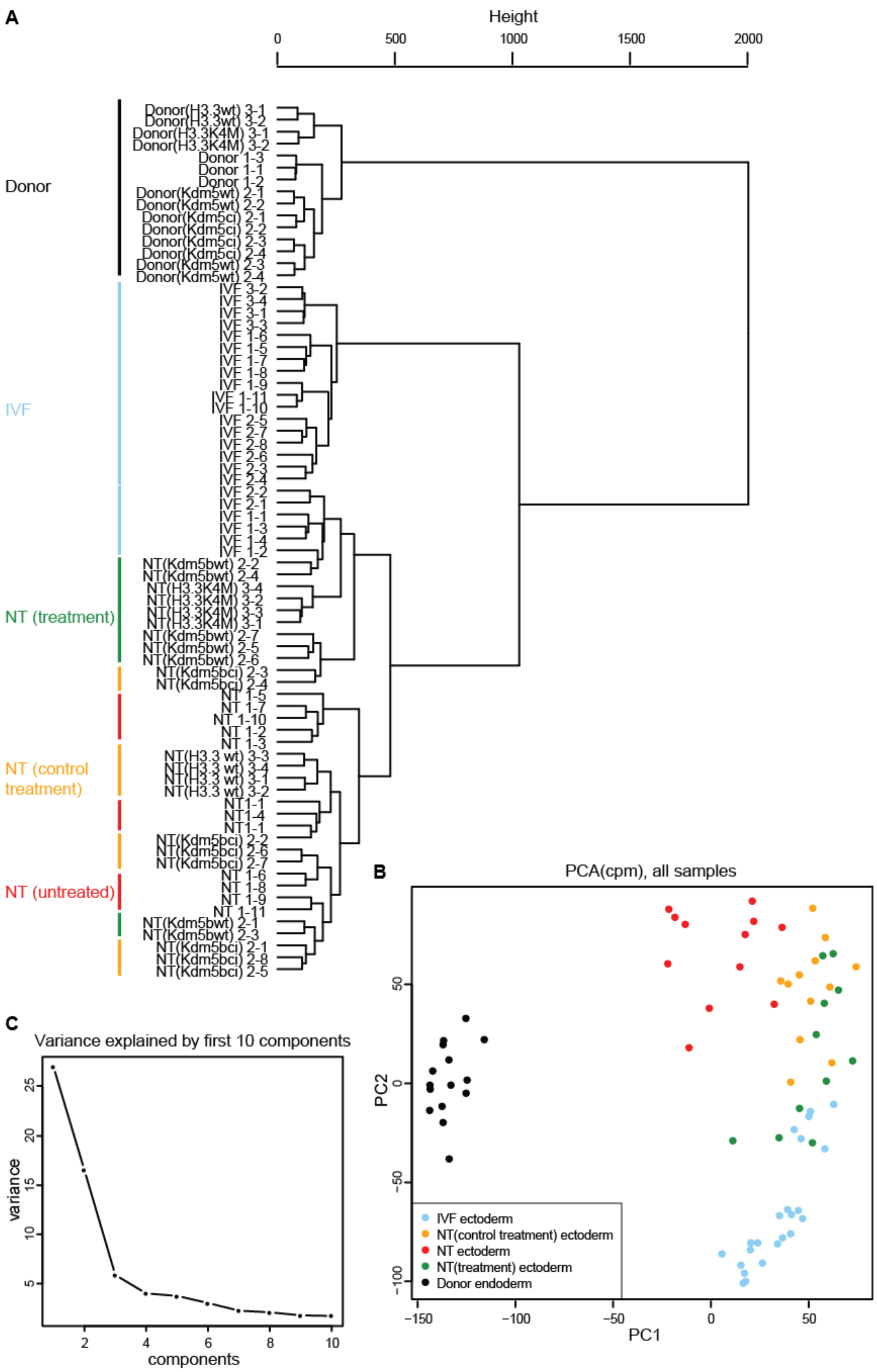

Figure S4. Related to Fig.1-4; Experimental variability analysis of RNAseq data presented in this study. (A) hierarchical clustering analysis performed on all experiments, filtered and normalized data. Colors codify the nature of the experiments: Donor cells, black; IVF, blue; NT

treatment (Kdm5b^{wt} or H3.3^{K4M}), green; NT control (Kdm5b^{ci} or H3.3^{wt}), orange; NT, red. In general, all RNA-seq experiments taken together group as expected into three classes (Donor, IVF and NT) irrespectively of the experimental batch. The hierarchical clustering was performed by using the Euclidean distance and ward.D linkage (as implemented in R). **(B)** First two principal components (which explain 27% and 16.5% of the variance, see panel (C)) of all experiments, filtered and normalized data. Also here, all RNA-seq experiments taken together group as expected into three classes (Donor, IVF and NT) irrespectively of the experimental batch. The PCA analysis was performed using the R function prcomp() using the parameter cor = T. Colors codify the nature of the experiments: Donor cells, black; IVF, blue; NT treatment(Kdm5b^{wt} or H3.3^{K4M}), green; NT control (Kdm5b^{ci} or H3.3^{wt}), orange; NT, red. **(C)** Percentage of variance explained by the first 10 components.

Table S1. Related to Fig.1, 2, 3, S3 and 4; Overview of biological replicates.

Differential gene expression analyses								
**Embryo (tissues) analysed by RNAseq**	**NT Experiments (Fig.1 and 2)**			**Kdm5b NT Experiments (Fig.3)**			**H3.3 NT Experiments (Fig.S4)**	
	**Exp.1**	**Exp.2**	**Exp.3**		**Exp.1**	**Exp.2**		**Exp.1**
**Donor (endoderm)**	1	1	1	**Kdm5b^{wt}**	2	2	**H3.3^{K4M}**	2
				**Kdm5b^{ci}**	2	2	**H3.3^{wt}**	2
**NT (ectoderm)**	3	4	5	**Kdm5b^{wt}**	4	3	**H3.3^{K4M}**	4
				**Kdm5b^{ci}**	4	4	**H3.3^{wt}**	4
**IVF (ectoderm)**	3	4	4		4	4		4

H3K4 demethylation in donor nuclei improves embryonic development of NT-embryos (results of quantifications)			
**Developmental stage**	**NT (Kdm5b^{wt})**	**NT (Kdm5b^{ci})**	**IVF**
**NT**	677	687	N/A
**Cleaved (stage 10)**	56 (100%)	69 (100%)	152 (100%)
**Gastrulae (stage 11)**	54 (97%)	67 (97%)	152 (100%)
**Neurulae (stage 21)**	42 (79%)	50 (70%)	140 (92%)
**Feeding tadpole (stage 45)**	34 (60%)	24 (33%)	127 (84%)

Donor, Endoderm donor cells; NT, nuclear transfer embryo; IVF, *in vitro* fertilized embryo; Exp., experiment; Numbers represent the number of tissues sequenced individidually, not in pools. 1 tissue was harvested from 1 embryo.

Table S2. Related to Fig.1 and 2; Overview of gene expression changes following nuclear transfer.

Categories	number of transcripts	% of total number of transcripts	Filters applied
**total number of transcripts**	24215	100%	CPM>1 in either all of the Donor- or 8 of IVF- or 8 of NT- samples.
**DE Donor and IVF**	17587	72.6%	FDR$^{Donor/IVF}$<0.05
**DE Donor and IVF and DE NT and IVF**	4504	18.6%	FDR $^{Donor/IVF}$<0.05 & FDR$^{NT/IVF}$ <0.05
**ON-Memory** (down-regulated in IVF vs Donor, up-regulated in NT vs IVF)	1534	6.3%	FDR$^{Donor/IVF}$<0.05 & logFC$^{Donor/IVF}$>0 & FDR$^{NT/IVF}$<0.05 & logFC$^{NT/IVF}$>0 & RPKM^{Donor}>1
**ON-Memory(3FC)** (down-regulated in IVF vs Donor, >3-fold up-regulated in NT vs IVF)	264	1.1%	FDR$^{Donor/IVF}$<0.05 & logFC$^{Donor/IVF}$>0 & FDR$^{NT/IVF}$<0.05 & logFC$^{NT/IVF}$>1.5 & RPKM^{Donor}>1
**OFF-Memory** (up-regulated in IVF vs Donor, down-regulated in NT vs IVF)	1346	5.6%	FDR$^{Donor/IVF}$<0.05 & logFC$^{Donor/IVF}$<0 & FDR$^{NT/IVF}$<0.05 & logFC$^{NT/IVF}$<0
**OFF-Memory(3FC)** (up-regulated in IVF vs Donor, >3-fold down-regulated in NT vs IVF)	88	0.36%	FDR$^{Donor/IVF}$<0.05 & logFC$^{Donor/IVF}$<0 & FDR$^{NT/IVF}$<0.05 & logFC$^{NT/IVF}$<-1.5
**Reprogrammed** (up- or down-regulated in IVF vs Donor, not DE between IVF and NT)	13083	54%	FDR$^{Donor/IVF}$<0.05 & exclude transcripts FDR$^{NT/IVF}$<0.05
**Reprogrammed-down** (down-regulated in IVF and NT vs Donor, not DE between IVF and NT)	6321	26.1%	FDR$^{Donor/IVF}$<0.05&logFC$^{Donor/IVF}$>0 &FDR$^{Donor/NT}$<0.05 & logFC$^{Donor/INT}$>0&RPKM^{Donor}>1&excludeFDR$^{NT/IVF}$<0.05
**Reprogrammed-up** (up-regulated in IVF and NT vs Donor, not DE between IVF and NT)	5501	22.7%	FDR$^{Donor/IVF}$<0.05 & logFC$^{Donor/IVF}$<0 &FDR$^{Donor/NT}$<0.05 & ogFC$^{Donor/IVF}$<0 & FDR$^{NT/IVF}$>0.05

Donor, Endoderm donor cells; NT, ectoderm of Nuclear transfer embryo, IVF, Ectoderm of *in vitro* fertilized embryo; DE, differentially expressed; FC, fold change; CPM, counts per million; FDR, false discovery rate; logFC, log2 fold change; RPKM, reads per kilobase per million;

Table S3. Related to Fig.3 and S3; Differential gene expression analysis of H3K4 demethylation experiments.

Overview of gene expression changes in H3K4 demethylated donor cells ($Kdm5b^{wt}$ or$H3.3^{K4M}$ mRNA injected) or control donor cells ($Kdm5b^{ci}$ or $H3.3^{wt}$ mRNA injected)

Categories	$Kdm5b^{ci}$ or $Kdm5b^{wt}$ treatment of donor cells		$H3.3^{wt}$ or $H3.3^{K4M}$ treatment of donor cells		Filters applied
	number of transcripts	% of ID transcripts	number of transcripts	% of ID transcripts	
**ID transcripts**	24758	100%	19210	100%	CPM>1 in either all of the donor, all IVF or all of NT samples
**DE Donor (treatment) vs Donor (control)**	102	0.4%	2953	15.4%	FDR<0.05
**total number of transcripts**	23318	99.6%	16257	84.6%	not DE in Donor($Kdm5b^{wt)}$ vs Donor($Kdm5b^{ci}$)

Overview of gene expression changes following nuclear transfer of H3K4 demethylated donor cells ($Kdm5b^{wt}$ or $H3.3^{K4M}$ treatment) or control donor cells ($Kdm5b^{ci}$ or $H3.3^{wt}$ treatment)

Categories	$Kdm5b^{ci}$ treatment of donor cells		$Kdm5b^{wt}$ treatment of donor cells		$H3.3^{wt}$ treatment of donor cells		$H3.3^{K4M}$ treatment of donor cells	
	number of transcripts	% of total transcripts	number of transcripts	% of total transcripts	number of transcripts	% of total transcripts	number of transcripts	% of total transcripts
**total number of transcripts**	23318	100%	23318	100%	16257	100%	16257	100%
**DE Donor and IVF**	15205	65.0%	15719	67.4%	13408	82.4%	13630	83.8%
**DE Donor and IVF and DE NT and IVF**	2359	10.1%	779	3.3%	4089	25.2%	4531	27.9%
**ON-Memory** (down-regulated in IVF vs Donor, up-regulated in NT vs IVF)	640	2.7%	307	1.3%	1169	7.2%	1317	8.1%
**ON-Memory(3FC)** (down-regulated in IVF vs Donor, >3-fold up-regulated in NT vs IVF)	231	1%	140	0.6%	194	1.2%	30	0.2%
**OFF-Memory** (up-regulated in IVF vs Donor, down-regulated in NT vs IVF)	796	3.4%	266	1.1%	1139	7.0%	1404	8.6%
**OFF-Memory(3FC)** (up-regulated in IVF vs Donor, >3-fold down-regulated in NT vs IVF)	183	0.8%	63	0.3%	72	0.4%	37.0	0.2%
**Reprogrammed** (up- or down-regulated in IVF vs Donor, and not DE in IVF vs NT)	10825	46.4%	11625	49.9%	9319	57.3%	9787	60.2%
**Reprogrammed-down** (down-regulated in IVF vs Donor, and not DE in IVF vs NT)	5666	24.3%	6024	25.8%	4560	28.0%	4645	28.6%
**Reprogrammed-up** (up-regulated in IVF vs Donor, and not DE in IVF vs NT)	5159	22.1%	5601	24.0%	4160	25.6%	3883	23.9%

ID, identified; Donor, Endoderm donor cells; NT, ectoderm of nuclear transfer embryo, IVF, Ectoderm of *in vitro* fertilized embryo; DE, differentially expressed; CPM, counts per million; FDR, false discovery rate; FC, fold change; For the filters applied, see Material and Methods.

Table S4; Related to STAR Methods; Primer table.

Gene expression analysis (Fig.S1 and S2)	
Name	Sequence
a2m-Fwd	GACGGTGCGCAAATATTTCC
a2m-Rev	AGCGTTCCCATCAGCATCTG
gata6-Fwd	CGATGCGTTCCCCTTCTG
gata6-Rev	ACAAGTCCACAGTTTTCATCAACAG
sox17β -Fwd	CGTCCTGGGCTGGAGATGT
sox17β-Rev	TCTCCTCTGGATTTGGCAGAA
foxA4-Fwd	TGTCCCCTCCTGGTGGAA
foxA4-Rev	TGGTGCCTCCCTGGAAGAC
darmin-Fwd	CCCCTGTGTCAGCTTGCAT
darmin-Rev	TGGGTGAAAATGAAACAGATTTGT
H4-Fwd	GACGCTGTCACCTACACCGAG
H4-Rev	CGCCGAAGCCGTAGAGAGTG
**ChIP analysis (Fig.S2)**	
gata6-Fwd-	CAAGTACTGGGAGCTGTACCACAA
gata6-Rev	AATTATGCTGCTAAGGGACAGACA
gata6-Fwd	CGGTGGTTGCGCGATATAG
gata6-Rev	CCAAGGAGCCATTGTGCAT
sox17β-Fwd	TCCCGCATCGCTCTTCAG
sox17β-Rev	TGGGCCGAACCCATGAC
sox17β-Fwd	GGGATGTTTGCACTTGGAAAG
sox17β-Rev	AGGAAGAAGCAGGTGAAGAGGAT
foxA4-Fwd	TGGACTCCAGAACATGCTAAATAGA
foxA4-Rev	TTGGTACATGGTATTCCAGTCCAT
foxA4-Fwd	TGTCCCCTCCTGGTGGAA
foxA4-Rev	TGGTGCCTCCCTGGAAGAC
darmin-Fwd	CCCCATGTGCCCCTAGCT
darmin-Rev	CAGTAGTAGCGCTTTTGAAGCAAA
darmin-Fwd	CAGTTGCCCCTTGCTCCAT
darmin-Rev	TGTCACAGACACACCGTGGTT

Fwd, Forward primer; Rev, reverse primer.
